# Abstracts of the ERICA ERN research conference

**DOI:** 10.1186/s13023-026-04450-y

**Published:** 2026-07-28

**Udine, Italy. 11-13 December 2024**

Publication of this supplement was funded by the EU H2020 CSA European Rare Disease Research Coordination and Support Action consortium (ERICA).

## A01: Robot-assisted one-stage combined anterior and posterior approach to sacral chordomas. A new frontier for multidisciplinary surgical treatment

Edoardo Ipponi^1^, Alfio Damiano Ruinato^1^, Federico Di Sacco^1^, Luca Morelli^2^, Rodolfo Capanna^1^, Lorenzo Andreani^1^

^1^University of Pisa - Department of Orthopedics and Trauma Surgery; ^2^University of Pisa - Department of Surgery

*Orphanet Journal of Rare Diseases 2026,* **21(1):**A01

**Introduction**

Chordomas are rare malignant mesenchymal tumors, accounting for 2-3% of all bone tumors, with an incidence of 0,1/100.000 years.

These neoplasms, which mainly involve the sacrum, generally have a slow progression and are frequently associated with late clinical presentation and diagnostic delays.

Chordoma is chemo-resistant and has a poor sensitivity to standard radiotherapy. Therefore, surgical treatment with partial or complete sacral resection still represents the mainstay of treatment. Sacral resection represents a challenging procedure even for the most experienced surgeon, with high rates of intra-operative and post-operative complications, including neurological lesions and impaired intestinal, urinary, or sexual functions.

Our study aimed to evaluate the effectiveness of a two-stage sacral resection combining an anterior intra-abdominal robotic approach and a posterior open approach to the sacrum.

**Materials and Methods**

Our study consists of a retrospective evaluation of all cases of sacral chordoma treated with a combined one-stage robotic anterior and open posterior approach in our institution between 2020 and 2023.

The diagnosis of chordoma was assessed with imaging evidence and confirmed by biopsy. All patients underwent MRIs and CT scans.

A multidisciplinary team treated all patients. The anterior robot-assisted resection was performed with the Da Vinci Xi System, which visualized and isolated the anterior surface of the sacrum and secured the noble structures of the inner pelvis for the later posterior resection. The tumor’s resection was then carried out with a posterior open approach.

Patients’Patients’ clinical status was assessed with periodical outpatient visits. Complications and local recurrences were recorded.

**Results**

Five cases were included in our study. The mean age of our patients was 66 years. All the patients had accidental diagnoses. Pain and constipation were the most common symptoms. No intraoperative complications occurred.

The resection was wide in four cases and marginal in one case; no intralesional resection was performed. The mean follow-up was 31 months. One patient suffered from bladder weakness; no other major post-operative complication was recorded. No local recurrence occurred, and all patients were CDF. Each case’scase’s sensitive symptoms had reduced after surgery.

**Conclusion**

The combined one-stage robotic anterior and open posterior approach represents a reliable treatment for sacral chordomas to maximize the quality of resection margins and minimize the risk of intra-operative and post-operative complications.

## A02: Harmonizing treatment and improving outcomes for pediatric adrenocortical tumors: a Pan-European prospective clinical trial by EXPeRT and ERN

M. Kuhlen^1^, C. Virgone^2^, G. Bisogno^3^, A. Ferrari^4^, E. Bien^5^, J. Roganovic^6^, Y. Reguerre^7^, J. Godzinski^8^, N. J. Farinha^9^, T. Ben-Ami^10^, F. Castinetti^11^, S. Lajic^12^, D. T. Schneider^13^, D. Orbach^14^, I. B. Brecht^15^, A. Redlich^16^

^1^Pediatrics and Adolescent Medicine, Faculty of Medicine, University of Augsburg, Augsburg, Germany; ERN PaedCanl; ^2^Pediatric Surgery, Department of Woman and Child’s Health, University Hospital of Padua, Padua, Italy; ^3^Pediatric Hematology-Oncology Division, University Hospital of Padua, Padua; ERN PaedCan; ^4^Pediatric Oncology Unit, Fondazione IRCCS Istituto Nazionale dei Tumori di Milano, Milan, Italy; ^5^Department of Pediatrics, Hematology and Oncology, Medical University of Gdansk, Gdansk, Poland; ERN PaedCan; ^6^Department of Pediatric Hematology and Oncology, Children´s Hospital Zagreb, Zagreb, Croatia; ^7^Department of Pediatric Hematology and Oncology, Félix Guyon University Hospital, St Denis, Réunion Island, France; ^8^Department of Pediatric Surgery, Marciniak Hospital, Wroclaw, Poland; Department of Pediatric Traumatology and Emergency Medicine, Medical University, Wroclaw, Poland; ERN PaedCan; ^9^Pediatric Oncology Unit, Centro Hospitalar Universitario Sao João, Porto, Portugal; ^10^Pediatric Hematology Unit, Kaplan Medical Center, Rehovot, Israel; ^11^Aix-Marseille Université, Institut National de la Santé et de la Recherche Médicale, Marseille Medical Genetics, Marseille, France; Endo-ERN, Adult Chair MTG4; ^12^Department of Women´s and Children´s Health, Division of Pediatrics, Unit for Pediatric Endocrinology and Metabolic Disorders, Karolinska Institutet/Karolinska University Hospital, Stockholm, Sweden; Endo-ERN, Pediatric Chair MTG1; ^13^Clinic of Pediatrics, Klinikum Dortmund, University Witten/Herdecke, Dortmund, Germany; ERN PaedCan; ^14^SIREDO Oncology Center (Care, Innovation and Research for Children, Adolescents and Young Adults with Cancer), Institut Curie, PSL University, Paris, France; ^15^Pediatric Hematology/Oncology, Department of Pediatrics, University Hospital Tuebingen, Tuebingen, Germany; ERN PaedCan; ^16^Department of Pediatrics, Pediatric Hematology/Oncology, Otto-von-Guericke-University, Magdeburg, Germany; Endo-ERN, Pediatric Chair MTG4

*Orphanet Journal of Rare Diseases 2026,* **21(1):**A02

**Introduction**

Pediatric adrenocortical tumors (pACTs) are extremely rare, accounting for approximately 0.2% of childhood cancers, yet they present significant clinical challenges due to their aggressive nature and heterogeneous presentation. Current treatment protocols across Europe vary widely, leading to inconsistent outcomes and fragmented understanding of the disease. The EXPeRT group [European Cooperative Study Group for Pediatric Rare Tumors in collaboration with the Endo-ERN proposes a Pan-European prospective clinical trial aimed at harmonizing treatment protocols, improving outcomes, and enhancing our understanding of pACTs.

**Objective**

This trial aims to establish a standardized treatment approach for pACTs across Europe, particularly focusing on high-risk patients with poor prognosis under current treatments. By creating a cohesive and collaborative research environment, we intend to gather comprehensive data that will inform evidence-based practices, ultimately improving survival rates and quality of life for affected children.

**Methods**

The trial will be conducted across multiple pediatric oncology centers in Europe, enrolling patients diagnosed with pACTs. Key components include.

1. *Standardization of Diagnostic and Treatment Protocols*: Uniform guidelines will be developed and implemented for the diagnosis, staging, and treatment of pACTs, ensuring consistency across participating centers.
2. *Stratification and Tailored Therapies*: Patients will be stratified based on factors such as tumor stage, genetic background, DNA methylation group, circulating tumor DNA, and clinical presentation. Treatment regimens will be tailored accordingly, with an emphasis on precision medicine.
3. *Data Collection and Analysis*: Systematic collection and analysis of data on patient demographics, treatment modalities, responses, and outcomes will be conducted. This will include molecular and genetic profiling to identify potential biomarkers for prognosis and therapeutic targets.
4. *Interdisciplinary Collaboration*: The trial will foster collaboration among oncologists, endocrinologists, surgeons, radiologists, pathologists, and geneticists to ensure a comprehensive approach to patient care and research.
5. *Patient and Family Support*: Psychosocial support for patients and their families will be prioritized, recognizing the profound impact of pACTs on quality of life.

**Expected Outcomes**: The trial aims to.

- Improve overall survival and disease-free survival rates for children with pACTs.
- Reduce variability in treatment outcomes by standardizing care practices.
- Enhance understanding of the biological basis of pACTs through genetic and molecular analysis.
- Establish a robust European database to inform future research and treatment innovations.

**Conclusion**

This Pan-European prospective clinical trial is a crucial step toward improving the management and outcomes of pACTs. By fostering collaboration and standardization, the EXPeRT group and ERN groups aim to pave the way for more effective treatments and improved quality of life for children with this rare and challenging disease.

## A03: Machine learning and Bayesian modeling of rare diseases

G. Penagos^1^

^1^Pontificia Universidad Javeriana, Bogotá, Colombia

*Orphanet Journal of Rare Diseases 2026,* **21(1):**A03

Orphan refers to medical products for conditions that are so infrequent that the cost for developing them in the pharmaceutical industry cannot be recovered by the expected sales. The aim is to ensure the patients suffering those conditions have access to suitable treatments and medical products. Research studies in rare diseases must assure quality, safety and effectiveness in medical products.

The small population size impairs conducting large trial designs, the standard statistical methodologies are inappropriate because the power of the test might not be sufficient for detecting a treatment effect. Among other challenges we found the heterogeneity induced by the phenotypic diversity, no access to natural history, the difficulties to generalize the results to a different population, the lack of reliable trial endpoints, and emerging ethical issues. In-silico models offer alternatives to deal with small populations, reducing costs and improving analysis.

The objective of this proposal is the development of mathematical models and the implementation of computer simulations to predict the efficacy of therapeutic molecules and related outputs as doses effects. We aim to establish a multidisciplinary approach, based on performance and credibility metrics, to assess computational models for designing small population clinical trials, and to support regulatory bodies for authorization assessment of orphan medicines.

To deal with these challenges and to overcome with statistical robustness, as test power, we will base the analysis on a hybrid methodology blending Artificial Intelligence (AI), in particular Machine Learning (ML), and Bayesian modelling, that allows the use of external information such as natural history, observational clinical studies, medical records, registries, pharmacovigilance and longitudinal studies. Two model approaches will be estimated, and their performance will be assessed using virtual patient cohorts, ML surrogates, and their agreement to use cases data of rare diseases.

First, a Bayesian parameter estimation to deal with small data availability will be incorporated into an ML algorithm. The posterior distributions can be used for inference, predictions and uncertainty assessment. On the ML side, some models are suitable for use with small data, e.g. SVM, and ensemble methods, e.g. random forest and XGBoost, that reduces variance and enhances accuracy. The second approach involves the use of Bayesian machine learning, e.g. Bayesian neural networks (BNN) estimated with Markov Chain Monte Carlo. This methodology allows treatment of small populations, incorporates prior knowledge, can perform model selection, delivers model uncertainty measures, and accounts for limitations in prediction accuracy allowing for informed decision-making.

The input variables: genotypes/phenotypes, disease characteristics/stage and/or clinical/surrogate endpoints, would be pre-processed to find the optimal subset of descriptors. Discarding non-informative variables makes the model interpretable and addresses computational burden, to this end, feature selection, evolutionary computation e.g. genetic algorithms, will be carried out.

## A04: Implementation and evaluation of clinical care structures for people with Differences of Sex Development (DSD) in Germany

Olaf Hiort^2^, Maike Schnoor^1^, Ulla Döhnert^2^, Martina Jürgensen^2^, Jannik Scherf^1^, Andreas Heidenreich^1^, Alexander Katalinic^1^ and DSDCare study group

^1^Institute of Social Medicine and Epidemiology, Lübeck, Germany; ^2^Division of Paediatric Endocrinology and Diabetes, Department of Paediatrics and Adolescent Medicine, University of Lübeck, Lübeck, Germany

*Orphanet Journal of Rare Diseases 2026,* **21(1):**A04

OH is paediatric chair and co-coordinator of Endo-ERN.

People with Differences of Sex Development (DSD) pose a challenge for healthcare. Lifelong holistic care requires a broad range of diagnostic approaches, qualified psychosocial, peer, fertility and sexual counselling, and structured therapeutic strategies including gonadal monitoring, hormone or surgical therapy. A quest has been taken to define centers of expertise for DSD and to substantiate and evaluate quality of care.

Based on the criteria defined by the European Reference Network (ERN) for Rare Endocrine Conditions, 10 health care providers in Germany qualify for specialized DSD care. Quality indicators (QI) were developed through a Donabedian model to define structure, process and outcome quality mainly based on the German national guideline “Differences of Sex Development”. Additionally, a consensus was reached though a structured process involving participating centers as well as patient organizations. Structural QIs are collected by an annual survey from the participating centers.

Patient-related process and outcome data are recorded in a central registry. Participants and in case of minors the legal guardians received a questionnaire including questions about satisfaction with care using the Y/CHC-SUN questionnaire. Medical and self-reported data were merged and analysed descriptively.

24 indicators for structural quality were consented, 7 for process and 8 for outcome quality. Indicators of process quality comprise e.g. diagnostic procedures, case conferences, peer counseling or patient education. For structural quality there was an increase of centers fulfilling the QIs in 14 items including e.g. team composition and qualification during the study period from 2020 until 2022. Still very few centers had fixed standard operating procedures e.g. for gonadal monitoring or long-term follow up after surgery. A total of 624 children, adolescents, and adults were registered. Of the 141 adults and 232 parents of children with DSD completing a questionnaire between May 2021 and December 2023, 81.9% of adults and 86.4% of parents stated to be “very” or “extremely satisfied” with their health care. Scores in the dimensions “doctor’s behaviour” and “patient-centred care” were very high for both adults and parents while the dimensions “clinical environment,”

“diagnosis/information,” and “coordination” were rated slightly lower. Some participants missed DSD training, psychological counselling, contact to self-advocacy groups and, in the case of adults, nutritional counselling.

Our approach allows for detailed evaluation of structural, process, and outcome quality. Health care insurers have secured appropriate funding for continuous evaluation of DSD care with centres organized in a “German Reference Network for Rare Conditions” and the maintenance of the registry. This will allow for sustainability of the benchmarking and improvement of health care provision for people with DSD nationally and can be used as a template for other countries.

Funding: The study was funded by the German Federal Ministry of Health, grant number 2519FSB503.

## A05: Supplementing published evidence with ‘expert evidence’: a pilot study using structured observation of cases from a clinical audit registry

Willemijn Irvine^1,2^, Olivia Spivack^1,2^, Charlotte Gaasterland^3,4,5^, Roel Bakx^2,6^, Carmen Mesas Burgos^2,7^, Alexandra Benachi^2,8^, René Wijnen^1,2^

^1^Department of Pediatric surgery, Erasmus MC Sophia Children’s Hospital, Rotterdam, The Netherlands; ^2^European Reference Network for Inherited Congenital gastrointestinal Anomalies (ERNICA), Department of Pediatric surgery, Erasmus MC Sophia Children’s Hospital, Rotterdam, The Netherlands; ^3^Knowledge Institute of the Dutch Association of Medical Specialists, Utrecht, The Netherlands; ^4^Amsterdam UMC, University of Amsterdam, Emma Children’s Hospital, Amsterdam, The Netherlands; ^5^European Reference Network on Rare Congenital Malformations and Rare Intellectual Disability ERN-ITHACA, Clinical Genetics Department, Robert Debré University Hospital, Paris, France; ^6^Department of Pediatric Surgery, Emma Children’s Hospital, Amsterdam UMC, University of Amsterdam and Vrije Universiteit Amsterdam, The Netherlands; ^7^Department of Pediatric Surgery, Karolinska University Hospital, Stockholm, Sweden; ^8^Department of Obstetrics and Gynecology, Antoine Béclère Hospital, Paris Saclay University, Clamart, France

**Correspondence:** Willemijn Irvine (w.irvine@erasmusmc.nl)

*Orphanet Journal of Rare Diseases 2026,* **21(1):**A05

**Introduction**

Developing high-quality guidelines for rare diseases has proven challenging. Sound guideline methodology can significantly contribute to the quality of clinical guidelines and provides structure to the development process. Most methodological strategies rely on the availability of high-quality evidence. However, as evidence is often lacking for rare diseases, many guidelines are developed based on expert opinion, lacking structured methodology and justification for their recommendations.

Exploring and evaluating innovative methodological strategies for rare disease guidelines is therefore imperative.

**Methods**

In this project, the use of clinical audit registry data as supplementary evidence will be piloted as an innovative approach to guideline development. This pilot will be applied during development of a European guideline on omphalocele, a rare birth defect which is under-researched, despite there being high practice variation across Europe. Members of the guideline development panel will be presented with registry data. They will be asked to rate the perceived efficacy of certain interventions on preselected outcomes using a likert-type scale ranging from benefit to harm. Experts’ perceptions will be summarized into so called ‘expert evidence’. The standard evidence-based Grading of Recommendations, Assessment, Development, and Evaluations (GRADE)approach to guideline development will be followed. Yet, during evidence synthesis, panel members will not only consider results from published evidence, but also ‘expert evidence’. Depending on the availability of evidence, one or both data sources will contribute to the development of recommendations following GRADE’s evidence to decision framework. This pilot will be evaluated in a cross-sectional study, assessing the added value of using ‘expert evidence’ considering the additional effort required by guideline panel members.

**Results**

This pilot study will result in a European guideline for omphalocele. By using insights from a clinical audit registry, the quality of recommendations may be improved. Besides, by evaluating additional value against invested effort, we will gain insight on the method’s potential to solve the problematic evidence gap present for so many rare diseases. A publication of the guideline, methodological description and evaluation is expected in the summer of 2025.

## A06: Achieving continuous quality improvement in a European rare disease network (Insert ERN): a conceptual framework

Olivia K. C Spivack^1^*, Willemijn F. E Irvine^1^*, Steffen Husby^2^, ERNICA representatives, Tomas Wester^3,4^, René M. H. Wijnen^1^

^1^Department of Pediatric Surgery, Erasmus MC Sophia Children’s Hospital, Rotterdam, the Netherlands; ^2^Department of Clinical Research, Faculty of Health Sciences, University of Southern Denmark; ^3^Department of Women’s and Children’s Health, Karolinska Institutet, Stockholm, Sweden; ^4^Unit of Pediatric Surgery, Karolinska University Hospital, Stockholm, Sweden

**Correspondence:** Olivia K.C Spivack (o.spivack@erasmusmc.nl)

* Joint first authors

*Orphanet Journal of Rare Diseases 2026,* **21(1):**A06

**Background**

The European Reference Network for […] is one of 24 European Reference Networks (ERNs) aiming to improve the quality of care for patients with rare and/or complex diseases. Bringing together 52 specialised hospitals from 21 countries across Europe, […] seeks to improve the quality of care for patients with […], regardless of where they live. The heterogeneity of its members/partners, however, both in regards to their organisational contexts and clinical practices, makes this a challenging endeavour. In attempt to mitigate these challenges, and foster a system of continuous quality improvement, we present the ‘[…] quality cycle’.

**Main body**

Structured based on the widely-used Plan-Do-Study-Act (PDSA) cycle, the […] quality cycle offers an iterative approach to quality improvement. It is made up of 5 steps: (1) Describing the desired level of care (2) Promoting guideline implementation (3) Measuring quality of care (4) Evaluating clinical practice (5) Conducting research.

**Conclusions**

The […] quality cycle offers a novel, structured approach to improving the quality of care for patients with […] across Europe. Evaluating this approach, and capturing learning points will be critical. This will be key to determining its transferability to other rare disease networks (ERNs), in support of our collective mission to ensure high-quality care is provided to all patients with rare diseases in Europe.

## A08: Obinutuzumab in rituximab-intolerant ANCA-associated vasculitis patients

J. R. van Leeuwen^1^, T. J. Rabelink^1^, Y. K. O. Teng^1^

^1^Center of Expertise for Lupus-, Vasculitis- and Complement-mediated Systemic diseases (LuVaCs), Department of Internal Medicine - Nephrology section, Leiden University Medical Center, Leiden, The Netherlands

ERNs: ERKNet and ERN-RITA

*Orphanet Journal of Rare Diseases 2026,* **21(1):**A08

**Introduction**

In anti-neutrophil cytoplasmic antibody (ANCA)-associated vasculitis (AAV) the fundament for both induction and maintenance treatment is anti-CD20 therapy with rituximab [1]. However, some patients experience serious infusion or allergic reactions, rendering them intolerant for rituximab retreatments and forcing them to rely on less effective alternatives such as azathioprine [1]. A logical alternative for rituximab is obinutuzumab of which equivalent or even superior effectiveness can be expected. Obinutuzumab is a novel, humanized type 2 anti-CD20 monoclonal antibody with demonstrated superiority to rituximab for treating lymphocytic leukemia and follicular lymphoma [2]. Also, superior B-cell cytotoxicity in vitro was demonstrated for rheumatoid arthritis and systemic lupus erythematosus [3]. Nevertheless, data on effectiveness of obinutuzumab in patients with AAV or other autoimmune disease are lacking and therefore obinutuzumab remains off-label for these indications. We now report on the immunological effects of obinutuzumab as alternative anti-CD20 therapy in AAV.

**Methods**

Six AAV patients who previously responded to rituximab, but developed rituximab intolerance, were studied in a case-control setting where patients were their own controls. Immunological effects on B-cell depletion (<10 × 10^6^ cells/Liter) and ANCA-course were compared. Only treatments where B-cell repopulation was measured during follow-up where included. Drug-related costs were determined based on costs for drugs and costs for drug-administration in the hospital.

**Results**

The immunological effects after 9 obinutuzumab and 8 rituximab cycles were compared. Obinutuzumab led to substantially longer B-cell depletion (median [interquartile range] of 13.7 [13.0-15.6] months) and more frequent ANCA-seroconversion (100%) than rituximab (7.2 [6.3-8.1] months and 38% respectively), the latter being in line with previous reported outcomes for a tailored rituximab regimen, i.e. 6.1 [3.1-9.2] months interval between retreatments and 44% ANCA-seroconversion [4]. Additionally, there were more decreasing ANCA-titers one year after obinutuzumab and also the duration of ANCA-negativity was longer after obinutuzumab (13.1 [10.4-19.5] months) than after rituximab (7.3 [7.1-13.5] months). Due to previous rituximab treatments, baseline ANCA-titers were lower for obinutuzumab (12.1 [9.0-20.4]) than rituximab (76.6 [37.1-106.3]). Noteworthy, the interval of obinutuzumab retreatments can be increased because of prolonged B-cell depletion. As such, when comparing drug-related costs to biosimilar-rituximab 6-monthly retreatments, obinutuzumab retreatments will be cost-effective at intervals of more than 7 months.

**Conclusion**

We can conclude that in rituximab-intolerant AAV patients obinutuzumab led to prolonged B-cell depletion and more frequent ANCA-seroconversion, both associated with reduced risk of relapse in AAV [1, 5]. Importantly, immunological effects of obinutuzumab were also superior to rituximab in AAV patients without rituximab-intolerance [4]. Our data suggest that obinutuzumab can be a relevant alternative for rituximab-intolerant AAV patients and potentially a cost-effective alternative for all AAV patients prompting the need for further studies.

**References**

1. Hellmich B, Sanchez-Alamo B, et al. EULAR recommendations for the management of ANCA-associated vasculitis: 2022 update. Ann Rheum Dis. 2023. 10.1136/ard-2022-223764.
2. Marcus R, Davies A, et al. Obinutuzumab for the First-Line Treatment of Follicular Lymphoma. N Engl J Med. 2017;377(14):1331-44.DOI: 10.1056/NEJMoa1614598.
3. Reddy V, Klein C, et al. Obinutuzumab induces superior B-cell cytotoxicity to rituximab in rheumatoid arthritis and systemic lupus erythematosus patient samples. Rheumatology (Oxford). 2017;56(7):1227-37.DOI: 10.1093/rheumatology/kex067.
4. Charles P, Terrier B, et al. Comparison of individually tailored versus fixed-schedule rituximab regimen to maintain ANCA-associated vasculitis remission: results of a multicentre, randomised controlled, phase III trial (MAINRITSAN2). Ann Rheum Dis. 2018;77(8):1143-9. 10.1136/annrheumdis-2017-212878.
5. van Dam LS, Dirikgil E, et al. PR3-ANCAs predict relapses in ANCA-associated vasculitis patients after rituximab. Nephrol Dial Transplant. 2021;36(8):1408-17. 10.1093/ndt/gfaa066.

## A09: European registries for rare endocrine and bone conditions: electronic tools for mapping and studying rare diseases

M. Cherenko^1, 2, 3^, A.L. Priego Zurita^1, 2, 3^, *T. M. de Rooij*^1, 2, 3^, S. F. Ahmed^1, 2, 4, 5^, N. M. Appelman-Dijkstra^1, 2, 3, 4^

^1^Department of Medicine, Division of Endocrinology, Leiden University Medical Centre – Leiden, NL; ^2^Endo-ERN; ^3^ERN BOND; ^4^University of Glasgow, Developmental Endocrinology Research Group, Royal Hospital for Children - Glasgow, UK; ^5^University of Glasgow, Office for Rare Conditions, Glasgow, UK

*Orphanet Journal of Rare Diseases 2026,* **21(1):**A09

**Introduction**

The European Registries for Rare Endocrine and Bone Conditions (EuRREB), formerly known as EuRRECa and EuRR-Bone, were created in collaboration with the European Reference Network on Rare Endocrine Conditions (Endo-ERN) and the European Reference Network on Rare Bone Diseases (ERN BOND) to comprehensively characterise Rare Conditions by facilitating data collection. The registries collect data using two web-based platforms: e-REC (e-Reporting of Rare Endocrine Conditions) and the Core Registry.

**Methods**

e-REC reporters were invited to register the number of new encounters via a monthly survey. Core Registry clinical contributors were invited to register new and existing cases and complete a set of Common Data Elements, generic patient-reported outcome measures (PROMs) and condition-specific modules. Patients can access the platform and complete outcomes. Both platforms collect conditions covered by Endo-ERN and ERN BOND, which are mapped according to the Orphanet classification.

**Results**

Until October 2024, 111 centres from 31 countries had reported on the e-REC platform. A total of 32,854 new cases in adults and 12,037 new cases in children were reported across nine main thematic groups (MTG). In children, conditions within the sex development and maturation group comprised 42% of all reported cases; among adults, pituitary conditions comprised 38% of all reported cases. Until October 2024, 3477 patients were added to the Core Registry by 48 centres from 20 countries. Distribution of cases: sex development and maturation 467 (13%), hypothalamic and pituitary 1187 (34%), calcium and phosphate 247 (7%), adrenal 254 (7%), thyroid 112 (3%), genetic endocrine tumour syndrome 56 (2%), growth and obesity 117 (3%), disorders of glucose and insulin metabolism 5 (0%), bone dysplasia 1032 (30%). PROMs have been completed by clinicians and patients with Eq. 5D being the most frequently used – 150 times (86 by patients and 64 by clinicians), BPI – 52 times, ICF-mobility – 43 times. Currently, the platform offers 11 condition-specific modules: pituitary tumour, parathyroid carcinoma, rare obesity, iPPSD/pseudohypoparathyroidism, rare hypophosphataemia, gender incongruence, aediatric differentiated thyroid carcinoma, osteogenesis imperfecta, achondroplasia, fibrous dysplasia/McCune-Albright syndrome (FD/MAS), and melorheostosis. The most active modules at the moment are FD/MAS and pituitary modules with 1406 and 1119 outcomes completed accordingly (several outcomes could be completed for one patient). An ongoing collaboration with ERN-EuroBloodNet will result in a new condition-specific module covering endocrine and bone complications in patients with Langerhans cell histiocytosis.

**Conclusion**

Data collection through registries facilitates studying the natural history and long-term clinical outcomes of rare endocrine and bone conditions. A versatile Core Registry has evolved from collecting Common Data Elements to collecting condition-specific outcomes and supporting inter-ERN partnership.

## A11: The impact of allocation bias in rare disease clinical trials with multiple endpoints

S. Schoenen^1^, N. Heussen^1,2^, R. -D. Hilgers^1^

^1^Institute of Medical Statistics - RWTH Aachen University, Aachen, Germany (*presenting author*); ^2^Medical School – Sigmund Freud Private University, Vienna, Austria

*Orphanet Journal of Rare Diseases 2026,* **21(1):**A11

**Introduction**

Deﬁning a single primary endpoint in rare disease clinical trials is challenging due to the variability in clinical presentation, diverse phenotypes, and limited understanding of the disease’s natural history. To address this complexity, multiple endpoints can be used, as they aggregate information and provide a more comprehensive description of treatment effects while enhancing statistical power. Therefore, using multiple endpoints is a promising approach in rare disease research. A well-known issue in rare disease trials is the potential for allocation bias, often due to the lack of blinding. Allocation bias occurs when knowledge of previous group assignments inﬂuences the allocation of subsequent patients. As a result, patients with certain characteristics may be preferentially assigned to either the treatment or control group, systematically violating the structural equality of both groups, even in randomized clinical trials. The current research aims to quantify whether allocation bias affects randomization-based inference in trials with multiple endpoints.

**Methods**

We derived a biasing policy, that quantiﬁes the effects of allocation bias in two-arm parallel group trials with continuous multiple endpoints. Following the ICH E9 guidance [1], we conducted a simulation study using our biasing policy to evaluate the impact of allocation bias through type I error rates across various randomization procedures and testing methods, including i.e. Bonferroni and scoring tests, that analyze clinical trials with multiple endpoints. Special focus is on clinical trials with small sample sizes.

**Results**

Simulations demonstrate that allocation bias inﬂates the type I error rates, leading to incorrect test decisions and distorted trial results. Even minimal bias effects can cause the nominal 5% signiﬁcance level to be exceeded. The degree of inﬂation depends on the chosen randomization procedure, the strength of allocation bias effects, and the multiple testing approach.

**Conclusion**

Assessing the impact of allocation bias at the trial design stage and selecting a randomization procedure that reduces bias are crucial for improving the validity of trial results. This is of special interest, in trials with small sample sizes and impacts the use of multiple endpoints such as patient-centered outcome measures (PCOM’s). The developed methodology can guide the selection of bias-mitigating randomization procedures, contributing to more reliable and robust *trial designs. Moreover*,* the approach can be extended to enable bias-adjusted testing*, providing a way to correct for allocation bias and ensure more valid results in rare disease trials.

[1] ICH E9: Statistical principles for clinical trials (1998).

https://database.ich.org/sites/default/files/E9Guideline.pdf Accessed 28 Feb 2024.

## A12: Clinicopathological determinants of recurrence risk in resected typical and atypical carcinoids

G. Lamberti^1^, E. Andrini^1^, S. Stumpo^1^, A. Di Odoardo^1^, G. Ricco^1^, M. G. Formelli^1^, A. Zappi^1^, N. Daddi^2^, D. Campana^1,3^

^1^Department of Medical and Surgical Sciences (DIMEC), Alma Mater Studiorum – University of Bologna, Bologna, Italy; ^2^Department of Thoracic Surgery, IRCCS Azienda Ospedaliera Universitaria di Bologna, Bologna, Italy; ^3^Medical Oncology Unit, IRCCS Azienda Ospedaliero-Universitaria di Bologna, Bologna, Italy

*Orphanet Journal of Rare Diseases 2026,* **21(1):**A12

**Introduction**

Pulmonary neuroendocrine tumors (NETs) are rare neoplasms, accounting for 1-2% of all lung cancers, and include typical carcinoid (TC) and atypical carcinoid (AC). TC is usually indolent, while AC is more aggressive. Surgery is the primary treatment, but recurrence remains a concern, particularly in AC or locally advanced-stage disease. We sought to identify factors associated with recurrence after radical surgery in patients with pulmonary carcinoid.

**Patients and Methods**

This retrospective study analyzed data from patients who underwent surgery for a TC or AC between January 2005 and April 2024. Primary endpoint was disease-free survival (DFS), deﬁned as the time from surgery to relapse or death from any cause, whichever occurred ﬁrst. Patients with diZuse idiopathic pulmonary neuroendocrine cell hyperplasia (DIPNECH) were excluded from DFS analysis. Clinical and pathological factors, including tumor size, Ki-67 index, mitotic count, necrosis, and TNM stage, were collected. Kaplan-Meier survival curves and Cox regression were used to estimate DFS and identify recurrence risk predictors. Multivariate analysis adjusted for confounding variables.

**Results**

Among the 125 patients, radical surgery was performed in 107 patients (85.6%). The primary endpoint analysis included 91 patients (85.0%) after excluding 16 cases with DIPNECH: 66.0% were female, median age was 58 (range 14-76). Smoking data were available for 49 patients, 53.1% of whom were smokers. Of this cohort, 51.7% had typical carcinoids, and 48.3% had atypical carcinoids. Tumor necrosis was present in 21.6%, with a median tumor size of 20 mm. The median Ki-67 index was 5%, and the mitotic count ranged from 0 to 10 (median 1.9). The most common type of surgery was lobectomy (75.6%), 32 (29.9%) patients experienced recurrence. Overall, the median DFS was 281 months (95%CI: 38.4-523.6).

Patients with TC had a signiﬁcantly longer DFS compared to those with AC (median 401 vs. 59 months, *p*<0.001). Patients with T1/2 tumors had longer DFS compared to T3/4 ones (median not reached vs. 11 months, respectively; *p*<0.001). Similarly, patients without tumoral lymph node involvement had longer DFS than those with node metastases (not reached vs. 31 months, respectively; *p*<0.001). Univariate analysis identiﬁed several predictors of recurrence, including AC (HR: 17.08, *p*<0.001), necrosis (HR: 7.64, *p*<0.001), Ki-67 as a continuous variable (HR:1.20, *p*<0.001), mitotic count as a continuous variable (HR:1.34; *p*<0.001), and tumor size as a continuous variable (HR:1.04, *p*=0.011). After adjusting for confounding factors in multivariate analysis, Ki-67 (HR:1.22, 95%CI: 1.11-1.35; *p*=0.017) and tumor size (HR:1.05, 95%CI: 1.01-1.09; *p*=0.023) retained their independent association with DFS.

**Conclusion**

Ki-67 index and tumor size are independent predictors of DFS in patients with TC/AC. These ﬁndings have crucial implications for post-surgical surveillance and design of clinical trials investigating adjuvant treatment strategies.

## A13: Overcoming challenges in epilepsy clinical trials: **the role of European Consortium for Epilepsy Trials (ECET)**

Sébile Tchaicha^1,3^, Eugen Trinka^2,3^, Alexis Arzimanoglou^1,3^

^1^Hôpital Sant Joan de Déu, Barcelona, Spain ; ERN EpiCARE; ^2^Christian Doppler University Hospital, Paracelsus Medical University, Salzburg, Austria; ERN EpiCARE; ^3^European Consortium for Epilepsy Trials, Dundrum, Dublin, Ireland

*Orphanet Journal of Rare Diseases 2026,* **21(1):**A13

Despite the regular approval of numerous antiseizure medications (ASMs), many individuals with epilepsy syndromes continue to experience seizures, suffer from comorbidities or may experience adverse events. While new treatments are urgently needed, they must first demonstrate safety and efficacy through rigorous clinical trials (CTs) to be approved by regulatory agencies. There remains a critical need for well-powered and representative clinical trials to develop novel treatments that can enhance quality of life, reduce seizure burden, minimize adverse effects, lower healthcare costs, reduce the risk of sudden unexpected death in epilepsy (SUDEP), and ultimately modify the natural evolution of the underlying aetiologies.

Several challenges persist in the design and execution of epilepsy CTs. The design and eligibility criteria have remained largely unchanged for decades, hindering site and participant recruitment, logistical efficiency, and efficacy assessment. Confusion between ASMs and disease modifiers, along with insufficient consideration of seizure types and syndromes, often leads to unspecific trial designs. Trials are frequently conducted in randomly selected epilepsy types, particularly in children, resulting in costly and heterogeneous patient pools. Furthermore, designs for rare disorders often require numerous trial sites and short observation periods, increasing heterogeneity of patients included. Additional barriers include the randomness of seizures, difficulties in accurately reporting seizure patterns, small effect sizes, and training requirements for caregivers and patients that are not applicable to real life. Feasibility studies, usually performed by CROs, also tend to overlook the specific characteristics of the epilepsy being studied.

Tackling these challenges requires strong international collaboration among expert investigators to promote high-quality clinical trials. Launched by the regional Executive Committee of the International League Against Epilepsy (ILAE-Europe) and endorsed by the ERN EpiCARE, the European Consortium for Epilepsy Trials (ECET) is a legal entity, established to elevate the standard of epilepsy trials - on medical therapies, devices and epilepsy-surgery - across Europe, focusing on both adult and paediatric populations. Experts from nearly 70 centres of reference in epilepsy care are already involved. ECET’s efforts include optimizing trial design and accelerating research by offering services to academia and industry, such as expert advice on trial design and implementation, a centralized and standardized adjudication process, and organizing educational activities to enhance the skills of researchers and healthcare professionals. The ERN EpiCARE Research Council and the WG on Clinical Trials and Targeted Therapies are the driving forces of ECET in developing long-term research strategies and in facilitating targeted investigations that strengthen connections between academia, industry, regulatory bodies, and patients.

The missions and already achieved actions of ECET will be presented, together with the benefits and limits, for the ERNs, of this model of action.

## A14: Shaping the future of epilepsy research in Europe: the ERN EpiCARE priorities

Alexis Arzimanoglou^1,2^, Sébile Tchaicha^1,2^, Helen Cross^3^, Kees Braun^4^, the ERN EpiCARE Research Council and Executive Commitee^1^

^1^Hôpital Sant Joan de Déu, Barcelona, Spain ; ERN EpiCARE; ^2^European Consortium for Epilepsy Trials, Dundrum, Dublin, Ireland; ^3^UCL GOSH Institute of Child Health, London, UK; ILAE President; ^4^University Medical Center Utrecht, Utrecht, Netherlands; ERN EpiCARE

*Orphanet Journal of Rare Diseases 2026,* **21(1):**A14

Epilepsy, a condition resulting from various acquired and genetic disorders, stands as one of the most prevalent serious neurological conditions, affecting over 70 million people globally [1]. Characterized by a predisposition to unprovoked seizure activity arising from abnormalities within cortical networks, epilepsy continues to pose significant health challenges. In Europe alone, more than 200,000 new cases are reported annually, equating to one new case every minute. Notably, 100,000 children and adolescents, as well as 130,000 individuals aged 65 years or older, are diagnosed with epilepsy each year [2, 3]. The mortality rate among individuals with epilepsy is 2 to 3 times higher than that of the general population [4].

In response to the above challenges, the ERN EpiCARE Coordination, along with the ERN EpiCARE Research Council (ERC) and a network of experts, established a list of priorities identifying critical needs across six key areas:

1. **Prevention of epileptogenesis and disease modification**.
2. **Genetics and targeted therapies**.
3. **Improved surgical decision-making**.
4. **Innovative trial design and outcome measures**.
5. **Artificial intelligence (AI) technologies for improving diagnosis and prediction**.
6. **Understanding and preventing comorbidities and mortality**.

In continuity with previous plans for epilepsy research (EPICLUSTER [5]), the present initiative seeks to identify and mitigate causes and risk factors, deepen understanding of epileptogenesis mechanisms, and promote the discovery of biomarkers and targeted treatments. To enhance, together with researchers and patient advocates, knowledge of epilepsy’s causes and consequences while limiting the occurrence and impact of seizures, comorbidities, and treatment-related side effects. The ultimate goal being to achieve treatment of the causes of the epilepsies rather than the symptoms.

In the near future, more of the important genetic risk factors contributing to epilepsy will be identified in a substantial proportion of individuals with non-acquired epilepsy. Genetically resolved epilepsies will eventually be grouped into larger sets that share common underlying biological causes or pathways. A promising emerging field within these priorities is the integration of AI technologies, which hold significant potential for supporting epilepsy diagnosis and predicting seizures.

The 6 research priorities will serve as a guide to ERN EpiCARE members and supporting partners involved in ERDERA, for grant applications within the HORIZON Europe programme and other collaborative projects. In line with the WHO intersectoral global action plan [1] on “Epilepsy and other Neurological Disorders” they also serve as a reference for our inter-ERNs (ERN-RND; ERN-NMD; ITHACA; MetabERN) joint initiatives.

1. Grisold, W. ∙ Freedman, M. ∙ Gouider, R. ∙ et al., The Intersectoral Global Action Plan (IGAP): a unique opportunity for neurology across the globe, J Neurol Sci. 2023; 449, 120,645.
2. Beghi E. The epidemiology of epilepsy. Neuroepidemiology. 2020;54(2):185–91.
3. Forsgren L, Beghi E, Oun A, et al. The epidemiology of epilepsy in Europe – a systematic review. *Eur J Neurol*. 2005; 12: 245–253.
4. Hitiris N, Mohanraj R, Norrie J, et al. Mortality in epilepsy. *Epilepsy Behav* 2007; 10: 363–376.
5. Henshall DC, Arzimanoglou A, Dedeurwaerdere S, et al. Shaping the future of European epilepsy research: Final meeting report from EPICLUSTER. Epilepsy Res. 2023;189:107068.

## A15: Evaluating the Performance of Pan Ran’s nomogram for predicting recurrence in GIST: an analysis of the Italian population

M. Grassi^1^, M. Ponzano^2^, M. Fedriga^2^, L. Mastracci^3^, F. Catalano^1^, V. Murianni^1^, S. Puglisi^1^, M. Cremante^1^, C. Pirrone^1^, A. Damassi^1^, L. Antonj^1^, M. Mascherini^4^, F. De Cian^4^, S. Mammoliti^1^, D. Comandini^1^

^1^Medical Oncology Unit 1, IRCCS Ospedale Policlinico San Martino, Genova, Italy; ^2^Department of Health Sciences (DISSAL), IRCCS Ospedale Policlinico San Martino, Genova, Italy; ^3^Dipartimento Scienze chirurgiche e diagnostiche integrate, Università degli Studi di Genova, Genova, Italy; ^4^Department of surgery, IRCCS Ospedale Policlinico San Martino, Genova, Italy

**ERN of our Centre**: Digestive tract, Endocrine organs, Female genital organs and placenta, Male genital organs and the urinary tract, Neuroendocrine system, Thorax.

*Orphanet Journal of Rare Diseases 2026,* **21(1):**A15

**Background**

GISTs are a rare entity but the most common malignant gastrointestinal mesenchymal tumors. About 50% of GISTs are diagnosed with localized disease and the recognition of the relapse risk is essential for the management [1,2,3]. Different risk classification systems have been developed [4,5,6,7,8]. Pan Ran et al. created a new nomogram for the Relapse Free Survival including new variables: DOG-1 positivity, gender and the effect of adjuvant therapy [9]. This new nomogram was developed and tested only in the Chinese population, but it is well known there are biological differences between Asian and Caucasian patients [10]. Therefore, we tested the performance of this newly developed nomogram in the Italian population.

**Methods**

We retrospectively analyzed the data of consecutive patients with primary localized GISTs underwent surgery at IRCCS San Martino Policlinic Hospital from 1995 to 2023. The prognostic index (PI) was calculated based on the Cox coeffcients reported. Univariable Cox PH models were performed in the validation dataset for all the variables included in the PI and variableswith a p-value<0.10 were included in the multivariable. Then a Cox model was run including the PI. C-index and Gonen and Heller Index were derived. Model fit in the validation set was assessed by running a Cox regression with the PI as an offset variable. The C-index and ROC curves were derived to measure discrimination and compare the performance with other commonly used prognostic tools.

**Results**

A total of 103 patients were included. At 5 years variables associated to events in the univariable models were mitotic count (5-10 vs. <5: HR=9.87, *p*=0.006; >10 vs. <5: HR=15.41, *p*=0.001) and tumor size (5-unit incr.: HR: 1.62, *p*=0.015). Mitotic count was significant in the multivariable model (p-values: 0.013 and 0.001). The PI was significantly associated with time to event at 5-years (PI 1-unit increase: HR=1.88, *p*=0.003) and c-index and Gonen and Heller Index were respectively 0.67 and 0.71. However, the PI showed a lack of fit in the validation dataset (*p*=0.016). At 5 years the C-indexes were 0.67 Pan Ran vs. 0.87 Joensuu, 0.84 Miettinen, 0.86 MSKCC. ROC AUC were Pan Ran 0.68, Joensuu 0.88, Miettinen 0.88, MSKCC 0.87.

**Conclusions**

Pan Ran’s nomogram has a lower discriminating ability than the existing prognostic scores in the Italian population. However, some of its variables such as the use of adjuvant therapy, are promising and could be integrated in future prognostic scores.

**References**

1. Miettinen M, Lasota J. Gastrointestinal Stromal Tumors: Review on Morphology, Molecular.

Pathology, Prognosis, and Differential Diagnosis. Arch Pathol Lab Med. 2006;130(10):1466-1478. 10.5858/2006-130-1466-GSTROM.

2. Rubin BP, Heinrich MC, Corless CL. Gastrointestinal stromal tumour. The Lancet. 2007;369(9574):1731-1741. 10.1016/S0140-6736(07)60780-6.
3. El-Menyar A, Mekkodathil A, Al-Thani H. Diagnosis and management of gastrointestinal stromal tumors: An up-to-date literature review. J Cancer Res Ther. 2017;13(6):889-900. 10.4103/0973-1482.177499.
4. Miettinen M, Lasota J. Gastrointestinal stromal tumors: pathology and prognosis at different sites. Semin Diagn Pathol. 2006;23(2):70-83. 10.1053/j.semdp.2006.09.001.
5. Gold JS, Gönen M, Gutiérrez A, et al. Development and validation of a prognostic nomogram for recurrence-free survival after complete surgical resection of localised primary gastrointestinal stromal tumour: a retrospective analysis. Lancet Oncol. 2009;10(11):1045-1052. 10.1016/S1470-2045(09)70242-6.
6. Chok AY, Goh BKP, Koh YX, et al. Validation of the MSKCC Gastrointestinal Stromal Tumor Nomogram and Comparison with Other Prognostication Systems: Single-Institution Experience with 289 Patients. Ann Surg Oncol. 2015;22(11):3597-3605. 10.1245/s10434-015-4400-z.
7. Gastrointestinal Stromal Tumor Nomogram: Survival Without Recurrence Following Surgery | Memorial Sloan Kettering Cancer Center. Accessed February 25, 2024. https://www.mskcc.org/nomograms/gastrointestinal/stromal_tumor.

8. Joensuu H, Vehtari A, Riihimäki J, et al. Risk of recurrence of gastrointestinal stromal tumour after surgery: an analysis of pooled population-based cohorts. Lancet Oncol. 2012;13(3):265-274. 10.1016/S1470-2045(11)70299-6.
9. Ran P, Tan T, Zhou H, et al. Nomogram for Predicting Recurrence-Free Survival of Primary Localized Gastrointestinal Stromal Tumor. J Pers Med. 2023;13(3):498. 10.3390/jpm13030498.
10. Zhang X, Ning L, Hu Y, et al. Prognostic Factors for Primary Localized Gastrointestinal Stromal Tumors After Radical Resection: Shandong Gastrointestinal Surgery Study Group, Study 1201. Ann Surg Oncol. 2020;27(8):2812-2821. 10.1245/s10434-020-08244-9.

## A16: A trial of Capecitabine and Temozolomide in neUroendocrine neoplAsms of the Lung – ACTUAL study

E. Andrini^1^, G. Lamberti^1^, S. Stumpo^1^, A. Di Odoardo^1^, G. Ricco^1^, M. G. Formelli^1^, A. Zappi^1^, D. Campana^1,2^

^1^Department of Medical and Surgical Sciences (DIMEC), Alma Mater Studiorum – University of Bologna, Bologna, Italy; ^2^Medical Oncology Unit, IRCCS Azienda Ospedaliero-Universitaria di Bologna, Bologna, Italy

*Orphanet Journal of Rare Diseases 2026,* **21(1):**A16

**Introduction**

Typical carcinoids (TC) and atypical carcinoids (AC) are rare, well-differentiated neuroendocrine tumors of the lung, whose diagnosis and treatment are challenging. TC is generally associated with a favorable prognosis, while AC exhibits more aggressive characteristics and a higher risk of metastasis. To date, the available therapeutic options for metastatic disease are limited. Everolimus is currently the only approved drug for the treatment of these tumors, but its efficacy is low, with a radiological response rate (ORR) of 2%. Temozolomide is associated with an ORR of 7.5-14% and it can be prescribed for the treatment of advanced TC/AC under L.648/1996. However, the combination of capecitabine and temozolomide (CAPTEM) has proven to be superior to temozolomide alone in the treatment of extrapulmonary neuroendocrine neoplasms, while retrospective studies in patients with TC/AC report ORRs of 18-30%.

**Patients and methods**

This is a prospective, multicenter, single-arm, phase II two-stage study according to Simon, enrolling chemotherapy-naïve patients with metastatic TC and AC of the lung. Prior treatment with somatostatin analogs or everolimus is allowed. Enrolled subjects will receive a combination of oral capecitabine 750 mg/m² twice daily (days 1-14) and temozolomide 200 mg/m² (days 10-14) every 28 days, until radiological progression, unacceptable toxicity, or patient refusal. The primary objective of the study is to evaluate the activity of CAPTEM in patients with TC/AC. Secondary objectives include efficacy and safety. The primary endpoint is the ORR according to RECIST v1.1, while secondary endpoints are progression-free survival (PFS), overall survival (OS), and toxicity, based on the frequency and severity of adverse events. The null hypothesis is that the ORR is <5%, while the alternative hypothesis is that the ORR is >5%. Assuming an alpha error of 10%, a beta error of 10%, and an ORR of 30% with CAPTEM, the study will enroll 7 patients in the first stage. If at least 1 response is observed, the study will proceed to the second stage, enrolling an additional 14 patients for a total of 21 patients. The study will be considered positive if at least 3 responses are observed.

**Results**

Assuming an accrual rate equal to 14 patients/year across 15 national Centers, the study enrollment should be completed in 24 months.

**Conclusion**

The combination of capecitabine and temozolomide (CAPTEM) may offer better outcomes compared to currently approved drugs in the treatment of advanced pulmonary carcinoids.

## A17: Multimodal identification of a cohort of patients with rare head and neck cancers within a university hospital clinical data warehouse (CDW)

A. La Rosa^1, 2^, M. Verdoux^3^, P. Riebler^1, 2^, I. Lolli^3^, X. Tannier^1^, B. Baujat^2^, E. Kempf^1, 4^

^1^Sorbonne University, Inserm, Université Sorbonne Paris-Nord, LIMICS, Paris, France; ^2^Assistance Publique - Hôpitaux de Paris, Sorbonne Université, GHU Tenon, Département d’ORL et chirurgie cervico-faciale, Paris, France (ERN : ERN EURACAN); ^3^Assistance Publique - Hôpitaux de Paris, GHU Paris Saclay, Unité de Recherche Clinique, Le Kremlin-Bicêtre, France; ^4^Assistance Publique - Hôpitaux de Paris, UPEC, GHU Henri Mondor, Département d’Oncologie Médicale, Créteil, France

*Orphanet Journal of Rare Diseases 2026,* **21(1):**A17

**Abstract**

**Introduction**

10% of head and neck cancers (HNCs) differ from the common squamous cell carcinoma located in the upper aerodigestive tract. These rare HNCs can be rare by their histology as well as their anatomical location. The federation of CDWs holds potential for advancing our understanding of these pathologies. This study aimed to develop an algorithm to identify rare HNC patients in a university hospital CDW.

**Methods**

We performed a multicenter cross-sectional observational feasibility study on the clinical data warehouse of a conglomerate of 39 university hospitals. A multimodal algorithm was developed to identify rare HNC patients by integrating ICD-10 codes, ADICAP pathology codes, and free-text data from electronic health records’ (EHRs) pathology reports. Regular expressions and components from the EDS-NLP v0.7.4 library were used to extract key clinical variables. Algorithm performance was evaluated by an HNC expert using a validation set of manually annotated cases.

**Results**

Among 333,852 cancer patients, 9,141 were identified as having HNC based on ICD-10 and ADICAP codes. Of these, 4,515 patients were classified as having rare HNC based on rare topography or rare histology, with a subset of 2,168 patients identified by at least two data sources forming the “consolidated rare HNC cohort.” The multimodal algorithm demonstrated a sensitivity of 91% and a specificity of 95% when relying on multiple data sources, with better performance observed for rare histology identification compared to rare topography.

**Discussion**

Our results suggest that combining structured codes and NLP-processed free-text data enhances the accuracy of patient identification in rare HNC cohorts. The inclusion of multiple data sources significantly improves performance metrics, especially in identifying rare histologies.

However, the algorithm’s effectiveness may be limited by the availability and quality of EHR data. Future work could focus on improving data quality and exploring the use of other data sources or advanced NLP models to enhance cohort identification in rare cancers.

**Conclusion**

This study demonstrates the feasibility and utility of a multimodal EHR-based approach to identify rare HNC patients. Incorporating free-text and structured data significantly improves cohort identification, offering a promising strategy for advancing research and clinical care in rare head and neck cancers.

## A18: Immunological-molecular profiling of chondrosarcoma (ChS)

Paweł Teterycz^1,11^, A. Zając^1^, A. Szumera-Ciećkiewicz^2^, J. Piątkowski^3^, J. Tuziak^2^, M. Wągrodzki^2^, A. Tysarowski^4^, E. Palmerini^5^, M. Gambarotti^6^, G. Frega^5^, M. Pierini^5^, A. Righi^6^, G. Magagnoli^6^, A. Dutour^7^, M. Jean-Denis^8^, T. Ibrahim^5^, J. -Y. Blay^9^, P. Golik^3,10^, A. Czarnecka^1^, P. Rutkowski^1^

^1^Department of Soft Tissue/Bone Sarcoma and Melanoma, Maria Sklodowska-Curie National Research Institute of Oncology, Warsaw, Poland; ^2^Department of Pathology, Maria Sklodowska-Curie National Research Institute of Oncology, Warsaw, Poland; ^3^Institute of Genetics and Biotechnology, Faculty of Biology, University of Warsaw, Warsaw, Poland; ^4^Cancer Molecular and Genetic Diagnostics Laboratory, Maria Sklodowska-Curie National Research Institute of Oncology, Warsaw, Poland; ^5^Osteoncology, Bone and Soft Tissue Sarcomas and Innovative Therapies, Istituto Ortopedico Rizzoli, Bologna, Italy; ^6^Anatomy and Pathological Histology, Istituto Ortopedico Rizzoli, Bologna, Italy; ^7^Translational Research Department, Centre Léon Bérard, Lyon, France; ^8^Department of Biopathology, Centre Léon Bérard, Lyon, France; ^9^Medicine Department, Centre Léon Bérard, Lyon, France; ^10^Institute of Biochemistry and Biophysics Polish Academy of Science, Warsaw, Poland; ^11^Department of Computational Oncology Maria Sklodowska-Curie National Research Institute of Oncology, Warsaw, Poland

*Orphanet Journal of Rare Diseases 2026,* **21(1):**A18

**Background**

There is still no effective treatment of ChS apart from surgery. ChS are considered as cold tumors with bad response to immunotherapy. However, immunophenotype of ChS and characterization of immune markers, as well as molecular background of this tumors are still poorly understood. The aim of this study was both immunological and molecular profiling of ChS and correlation these features with clinical outcomes. This study may also help in selection of patients (pts) who can benefit from targeted therapy.

**Methods**

We enrolled 99 pts diagnosed with primary ChS: 28 G1, 37 G2, 24 G3 and 10 dedifferentiated cases). For immune cells infiltrates evaluation, tissue microarrays of central and peripheral region of the tumors were prepared from formalin-fixed paraffin-embedded tissue blocks. Immunohistochemical analysis covered 20 markers defining effector T cells, antigen-presenting cells, and M1/M2 macrophages, T cells exhaustion, PD-L1 expression, et al. Molecular profiling and tumor mutational burden were obtained by next-generation sequencing of 409 genes. Additionally, microsatellite instability was evaluated. Multiparametric feature selection for this high-dimmentional data was performed using LASSO-based Cox regression.

**Results**

Median overall survival of all pts was 64.6 (95%CI: 44.2 -) months. We identified 3 different immunophenotypes among ChS pts which can be described as “cold”, “hot” and “intermediate”. The most frequently mutated genes (detected in >10% of pts) were *IDH1/2*, *TP53*, *TAF1*, and *RNF213.* The presence of *IDH1/2* or *TP53* mutation was significantly depended on tumor grade and these were observed more frequently in high grade ChS. In multivariate Cox analysis the presence of „hot” (HR: 3.36, CI:1.12-10.1, *p*<0.05) or „intermediate” phenotype (HR: 3.07, CI:1.01-9.4, *p*<0.05), and *IDH1* mutations (HR: 3.34, CI:1.56-7.2, *p*<0.01) were poor prognostic factors, as well as tumor grade and size.

**Conclusions**

Immune phenotypes related to higher immune cell infiltrates inside the tumor and occurrence of *IDH1* mutation are independent poor prognostic factors and predict worse outcomes in ChS pts. These finding indicated that it is possible to identify a group of pts with a poor prognosis, in whom the use of immunotherapy and inhibitors of IDH-mutant could potentially provide clinical benefit.

## A19: Patient perspective in the first German national registry for rare diseases (NARSE) within the FAIR4Rare evaluation project

C. Finis^1^, J. Schepers^2^

^1^German Osteogenesis Imperfecta Association (DOIG); European Patient Advocate of the ERN BOND; Berlin Institute of Health (BIH) at Charité, Berlin, Germany; ^2^Berlin Institute of Health (BIH) at Charité, Berlin, Germany

*Orphanet Journal of Rare Diseases 2026,* **21(1):**A19

**Introduction**

NARSE is being established by the Berlin Institute of Health at Charité with funds from the Federal Ministry of Education and Research and support from the Eva Luise and Horst Köhler Foundation and other parties. It works according to the FAIR principles. The aim of NARSE is to use a focused data set to gain initial insights into the epidemiology of as many rare diseases as possible, to improve research and translation of new therapies, to support the networking of those affected, and to promote their fairer participation in the healthcare system. The NARSE data set, like the RD module of the core data set of university medicine, is based on the Set of Common Data Elements of the European Rare Disease Registry Infrastructure (ERDRI CDS) to enable networking with European initiatives and ERN registries. In the ongoing FAIR4Rare project, which is funded by the self-governing body of the German healthcare system, project partners from health services research, economics, clinics and patient organizations are evaluating the NARSE.

**Patients and Methods**

The Fair4Rare evaluation is supporting the BIH registry operator in identifying and adjusting strengths and weaknesses as well as opportunities and threats regarding the goals of the new infrastructure (SWOT analysis), within the scope of economic possibilities. FAIR4Rare will be carried out with the participation of patient organizations. The situations of the diseases cystic fibrosis, osteogenesis imperfecta and genetic forms of obesity will be examined as examples. Each patient organization will contribute with its own initial situation. The Cystic Fibrosis Association already has its own German registry and is aligning it with NARSE. The DOIG e.V. and the ACHSE e.V. represent two diagnoses without their own registry. While the DOIG represents a larger group within RDs with OI, the ACHSE represents a smaller group within the field of RDs with genetic obesity.

Patient organizations can also address topics that are in their own interest. For example:

- What is the added value of NARSE compared to existing registries?
- From a patient perspective, what variables are missing to create a meaningful registry?
- How is the transparency requirement of the GDPR fulfilled?

The following points have been included in the specifications of the development and evaluation project. Patients to.

- Be able to view their entry,
- Be able to send messages regarding the entries to the registrants,
- Be involved in formulating questions and be able to introduce their own questions into projects,
- Be supported to maintain their own documentation.

**Conclusion**

NARSE and the accompanying FAIR4Rare evaluation incorporate the patient perspective in a meaningful and useful way for all stakeholders by involving patients.

## A20: Patient-centered perspective - insights from an EPAG workshop

I. Alves^1^, L. Casareto^2^, N. Z. Großmann^3^, G. Giordano^2^, C. Finis^5^, M. C. la Forgia^6^, M. Sessa^7^, R. T. Skarberg^8^, T. T. Sylvest^9^, L. Sangiorgi^2^

^1^ANDO Portugal; School of Health and Human Development, University of Évora – CHRC, ERN BOND ePAG; ^2^Department of Rare Skeletal Disorders, IRCCS Istituto Ortopedico Rizzoli, ERN BOND; ^3^FOP e.V; LOUDRARE e.V., Freie Universität Berlin, Charité - Universitätsmedizin Berlin, University of Pennsylvania, ERN BOND ePAG; ^4^DOIG – Deutsche Gesellschaft für Osteogenesis imperfecta (Glasknochen) – Betroffene e.V., ERN BOND ePAG; ^5^ACAR Aps Associazione Conto Alla Rovescia, ERN BOND ePAG; ^6^AISAC Associazione per l’Informazione e lo Studio dell’Acondroplasia ONLUS, ERN BOND ePAG; ^7^Osteogenesis Imperfecta Federation Europe (OIFE), ERN BOND ePAG; ^8^X-bunden Hypofosfatæmisk Rakitis (XLH), arvelig rakitis, ERN BOND ePAG

*Orphanet Journal of Rare Diseases 2026,* **21(1):**A20

**Abstract**

**Introduction**

Rare bone diseases (RBDs) present unique challenges requiring collaborative approaches between patients’ representatives, patient organisations, and multidisciplinary healthcare teams [1]. The European Patient Advocacy Groups (ePAGs) representatives of the European Reference Network on Rare Bone Diseases (ERN BOND) organised a workshop to approach patient priorities and unmet needs, aiming to guide future initiatives and improve quality of life for individuals with RBDs.

**Patients and methods**

Six ePAG representatives participated and presented in a hybrid workshop during the 2023 ERN BOND annual meeting. Discussions focused on four key areas: pregnancy in women with RBDs, transition from paediatric to adult care, movement analysis and mobility functional limitations, and pain management. Mentimeter surveys were used to collect and prioritize participant feedback. The workshop involved 38 participants from 7 countries, including healthcare professionals, patient representatives, and pharmaceutical companies’ stakeholders.

**Results**

The workshop highlighted critical unmet needs in RBD patient care. For pregnancy, participants emphasized the need for specialized care, evidence-based guidelines, and access to experienced healthcare professionals. Women with RBDs face additional risks during pregnancy, delivery and lactation, including breathing difficulties, miscarriage, anaesthesia procedures challenges, loss of bone mineral density, necessitating careful monitoring and support. Transition to adult care required a well-coordinated, structured program ensuring continuity of care and patient empowerment. Participants stressed the importance of educating healthcare professionals on multidisciplinary approaches and identifying a “patient manager” to facilitate team coordination. Movement analysis and mobility functional limitations demanded comprehensive assessments and tailored rehabilitation programs. Participants highlighted the need for gait analysis, assistive devices, and adaptive equipment to enhance mobility and independence. Pain management necessitated a multifaceted approach, incorporating patient-reported experiences and multidisciplinary team involvement. Effective pain treatment, reliable measuring tools, and increased awareness were identified as priorities.

**Conclusion**

This patient-centred workshop provided valuable insights into RBD patient priorities, guiding future ERN BOND initiatives. The identified themes - pregnancy care, transition programs, movement analysis, and pain management - represent critical areas where targeted interventions can significantly improve patient outcomes and quality of life. These findings underscore the importance of patient engagement in rare disease research and care, paving the way for more effective, person-centred solutions in RBD management. The workshop has already informed ERN BOND’s planning for the next project phase, including enhanced healthcare professional training, dedicated focus groups for pregnancy care including new variable included in ERN BOND registry (EuRR Bone), a scoping review methods for gait analysis, future gait analysis projects, and development of tailored transition models. By leveraging the ERN BOND network’s expertise and collaborating with patient representatives, these efforts are expected to significantly advance RBD management and patient outcomes across Europe.

1. Tumiene B, Graessner H, Mathijssen IM, Pereira AM, Schaefer F, Scarpa M, Blay JY, Dollfus H, Hoogerbrugge N. European Reference Networks: challenges and opportunities. J Community Genet. 2021;12(2):217–29.

## A21: Enhancing ERN-industry collaboration with the Together for Rare Diseases’ forum for exchange of information in research

Sheela Upadhyaya^1^, Victoria Hedley^1^, Mathieu Boudes^1^, Clara Romero^1^

^1^Together For Rare Diseases secretariat, Brussels, Belgium

*Orphanet Journal of Rare Diseases 2026,* **21(1):**A21

The European Reference Networks (ERNs) play a pivotal role in advancing rare disease research and care across Europe. While some ERNs have already established successful industry collaboration forums, there is a growing need for a comprehensive, pan-ERN approach to maximize impact and foster engagement with industry partners. Together For Rare Diseases proposes the establishment of a Forum for Exchange of Information to enhance ERN-Industry engagement, addressing the current gap in awareness and interaction between these key stakeholders.

The primary objective of this forum is to create a collaborative platform that facilitates exchanges between ERNs and industry, promoting mutual understanding and fostering joint projects. Several models for this forum are proposed, ranging from pan-ERN research conferences to ERN-specific research matchmaking events, building upon existing successful initiatives.

Key considerations for the forum may include:

- **Flexible formats**: Options include pan-ERN research conferences, ERN-Industry strategy forums, ERN-specific industry research forums, and research matchmaking events.
- **Strategic discussions**: The forum would provide opportunities for ERN and industry representatives, and patient advocates to discuss subjects of mutual interest from cross-disease perspectives.
- **Raising awareness**: Many companies, especially smaller biotech firms, lack knowledge about ERNs’ capabilities and priorities. Conversely, the forum would highlight industry’s contributions beyond funding, such as scientific and regulatory expertise, access to infrastructure and assets; operational capabilities and capacity; collaborative leadership and innovation drive; and research ecosystem strengthening.
- **Tailored approaches**: Subnetworks fora could be organized alongside existing ERN meetings or as standalone events, focusing on specific disease areas or research priorities.

Challenges to address are many and include regulatory and policy (perceived) limitations, ERNs’ lack of legal entity status, ERNs’ lack of resources (manpower and funding), ERNs’ different research and collaboration maturity, size and capacities and the need for clear policies on industry and ERNs. The forum’s success will depend on securing support from trade associations, identifying synergies with existing initiatives like ERDERA to avoiding duplication of efforts and demonstrating effective collaboration models through pilot projects.

This initiative aims to stimulate scientific collaborations between ERNs and industry, ultimately advancing rare disease research and improving patient care across Europe.

*Together for Rare Diseases (Together4RD) is an agile multi-stakeholder initiative aimed at supporting ERNs to collaborate with stakeholders to pursue opportunities that will address unmet medical needs of people living with rare diseases. It is funded by EFPIA*,* EUCOPE*,* Sanofi*,* Takeda*,* Novo Nordisk and UCB and governed by a multi-stakeholder steering group including representatives from ERNs*,* research communities*,* and patient representatives.*

## A22: Defining priorities in the transition from paediatric to adult healthcare for rare bone disease patients: a dialogic approach

Davide Scognamiglio^1,2^, M. Boarini^1,2^, M. C. la Forgia^2,3^, E. Grippa^2,3^, S. Forni^2,3^, A. Sergi^4^, A. Romeo^2,3^, G. Massa^2,3^, L. Sangiorgi^1,2^

^1^Department of Rare Skeletal Disorders, IRCCS Istituto Ortopedico Rizzoli, Bologna, Italy; ^2^European Reference Network on Rare Bone Diseases (ERN BOND), Bologna, Italy; ^3^A.C.A.R. Aps - Associazione Conto Alla Rovescia, Rome, Italy; ^4^SOC Monitoraggio e Programmazione Performance Clinico-assistenziale, Azienda USL Toscana Centro, Florence, Italy

**First Author:** Davide Scognamiglio (davide.scognamiglio@ior.it)

*Orphanet Journal of Rare Diseases 2026,* **21(1):**A22

**Abstract**:

**Introduction**

The transition from pediatric to adult healthcare for patients with rare bone diseases such as Multiple Osteochondromas (MO), Ollier Disease (OD), and Maffucci Syndrome (MS) poses significant challenges. This study, conducted during the 2023 meeting of the Italian patients association of MO, OD and MS, aimed to identify key priorities during this transition through a mixed-methods approach.

**Materials and methods**

A total of 115 individuals participated in an Open Dialogue Approach (ODA) activity, including patients (26.1%), caregivers (53%), healthcare professionals (14.8%), and healthy siblings (6.1%). Participants discussed their experiences and identified key priorities for improving healthcare transition. Following the ODA, 79 individuals (94.9% adults) completed a feedback survey that further refined these priorities. Data was analyzed through content analysis and priority rating.

**Results**

The main themes identified included coordination and continuity of care, patient empowerment, communication with healthcare professionals, social and practical support, and transition planning. Psychological support emerged as the most important need, with 92% of respondents rating it as a top priority. Additionally, 87% of participants emphasized the importance of listening to younger patients, while 86% highlighted the need for improved communication with empathetic clinicians and being integrated into a well-defined care plan. In contrast, transitional surgery and the avoidance of urgent procedures during transition were considered less important, receiving only 25% and 33% of the vote, respectively. Interestingly, adult patients prioritized psychological support (100%), whereas adolescents focused on improving communication with clinicians (100%). Healthcare professionals and caregivers also prioritized communication (93% and 86%, respectively), while healthy caregivers aligned more closely with the overall group preferences.

**Conclusion**

This study highlights the critical need for a well-structured, empathetic approach in the transition from pediatric to adult care for MO, OD, and MS patients. Coordination, communication, and psychological support are vital components. These findings offer a framework for developing a white paper on improving care for individuals with these rare conditions. The methodology used may serve as a model for other patient associations seeking to identify and address members’ needs.

## A23: Utility of polygenic risk score for prediction of dilated cardiomyopathy and modulation of severity of LV dysfunction among cases

D. R. Kramarenko^1,8^, S. J. Jurgens^1^, J. T. Ramo^2^, C. A. Van Orsouw^1^, J. J. Hottenga^4^, P. Charron^5^, B. Meder^6^, A. Palotie^2^, M. Daly^2^, P. T. Ellinor^7^, Y. M. Pinto^1^, K. G. Aragam^7^, S. N. Van Der Crabben^1^, A. S. Amin^1,8^, C. R. Bezzina^1^

^1^Amsterdam UMC - Location Academic Medical Center, Amsterdam, Netherlands (The); ^2^Institute for Molecular Medicine Finland (FIMM), Helsinki, Finland; ^3^Amsterdam University Medical Centre, Amsterdam, Netherlands (The); ^4^Vrije Universiteit Medical Centre (VUMC), Department of Biological Psychology, Amsterdam, Netherlands (The); ^5^Institute of Cardiometabolism and Nutrition - ICAN, Paris, France; ^6^University Hospital Heidelberg, Department of Medicine III, Heidelberg, Germany; ^7^Broad Institute, Cardiovascular Disease Initiative, Cambridge, USA; ^8^ERN-GUARD Heart, Amsterdam, Netherlands

*Orphanet Journal of Rare Diseases 2026,* **21(1):**A23

**Abstract**:

**Background**

Dilated cardiomyopathy (DCM) is a predominant cause of heart failure and a leading indication for cardiac transplantation, yet its aetiology remains incompletely elucidated. While rare genetic variants are established as causative factors in familial forms of the disease, recent genome-wide association studies (GWAS) have also underscored the signiﬁcant contribution of common genetic variation to DCM. Polygenic scores (PGS) constructed from GWAS have demonstrated promise in risk stratiﬁcation for a range of common diseases and may similarly have value for risk prediction in DCM. However, data regarding the clinical utility of PGS in DCM remains sparse.

**Purpose**

We aimed to evaluate the utility of PGS for prediction of DCM and its association with left ventricular ejection fraction (LVEF) and family history. We further aimed to assess the contribution of PGS in DCM patients with established causative rare variants (genotype-positive) and those without (genotype-negative).

**Methods**

We used a previously-constructed PGS, which was developed from a GWAS meta-analysis (8387 DCM cases and 939161 controls) and multi-trait analysis (MTAG) with cardiac MRI traits. In the present analysis, we calculated PGS for a newly assembled cohort of 978 DCM cases sourced from a major university medical centre and 7207 controls from a national register. To evaluate the clinical utility of PGS, we built logistic regression models and assessed the association between PGS and DCM status. Subsequently, we performed subgroup analyses, including: (i) individuals of European ancestry, (ii) non-European ancestry, (iii) males, (iv) females, (v) genotype-positive participants(*n*=193), and (vi) genotype-negative participants (*n*=294). We then used linear and logistic regression models, respectively, to assess the associations of PGS with LVEF and documented family history of DCM.

**Results**

**Fig. 1:**
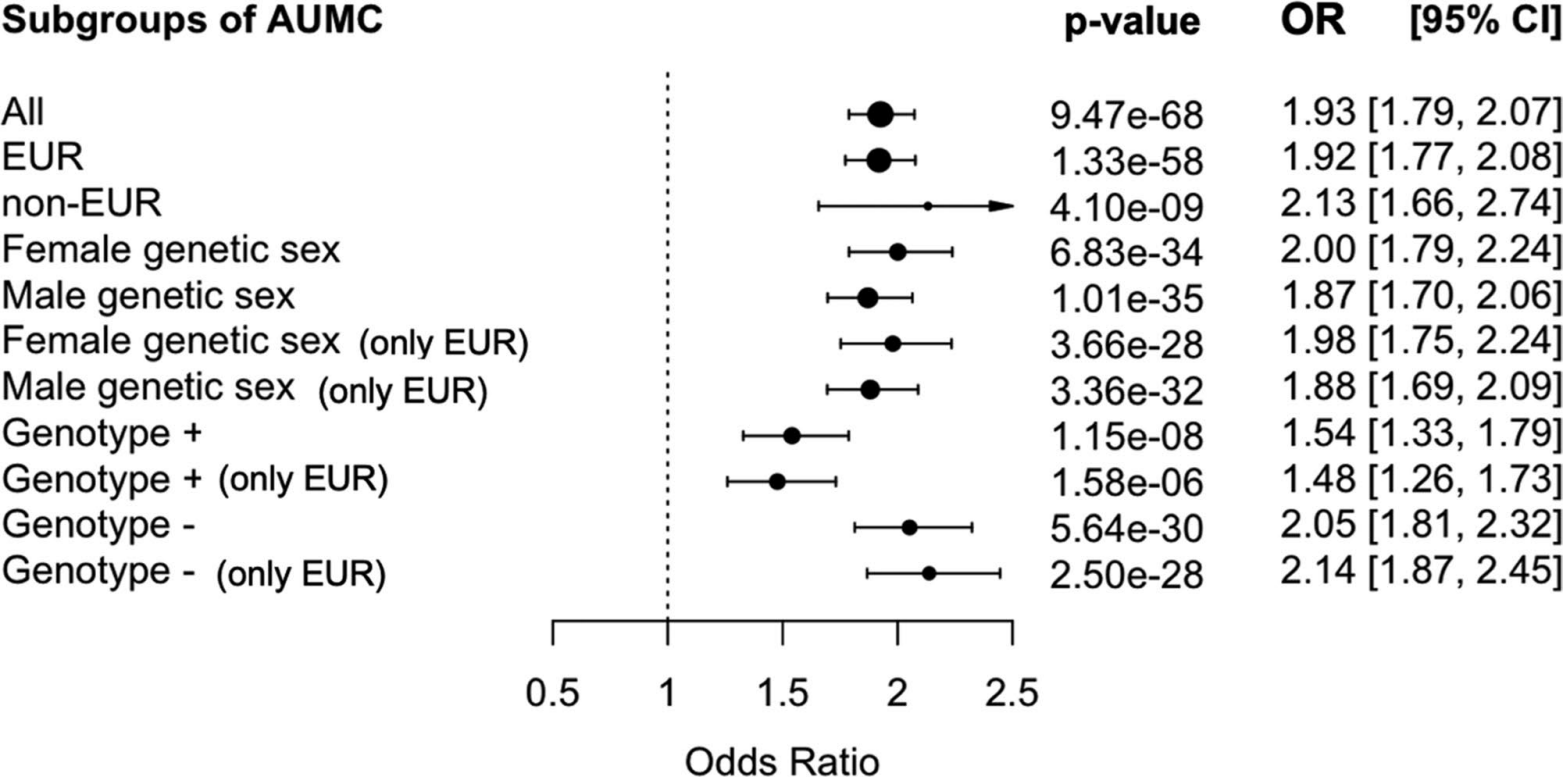
Association of PGS with DCM in all AUMC patients and specific subgroups

**Fig. 2:**
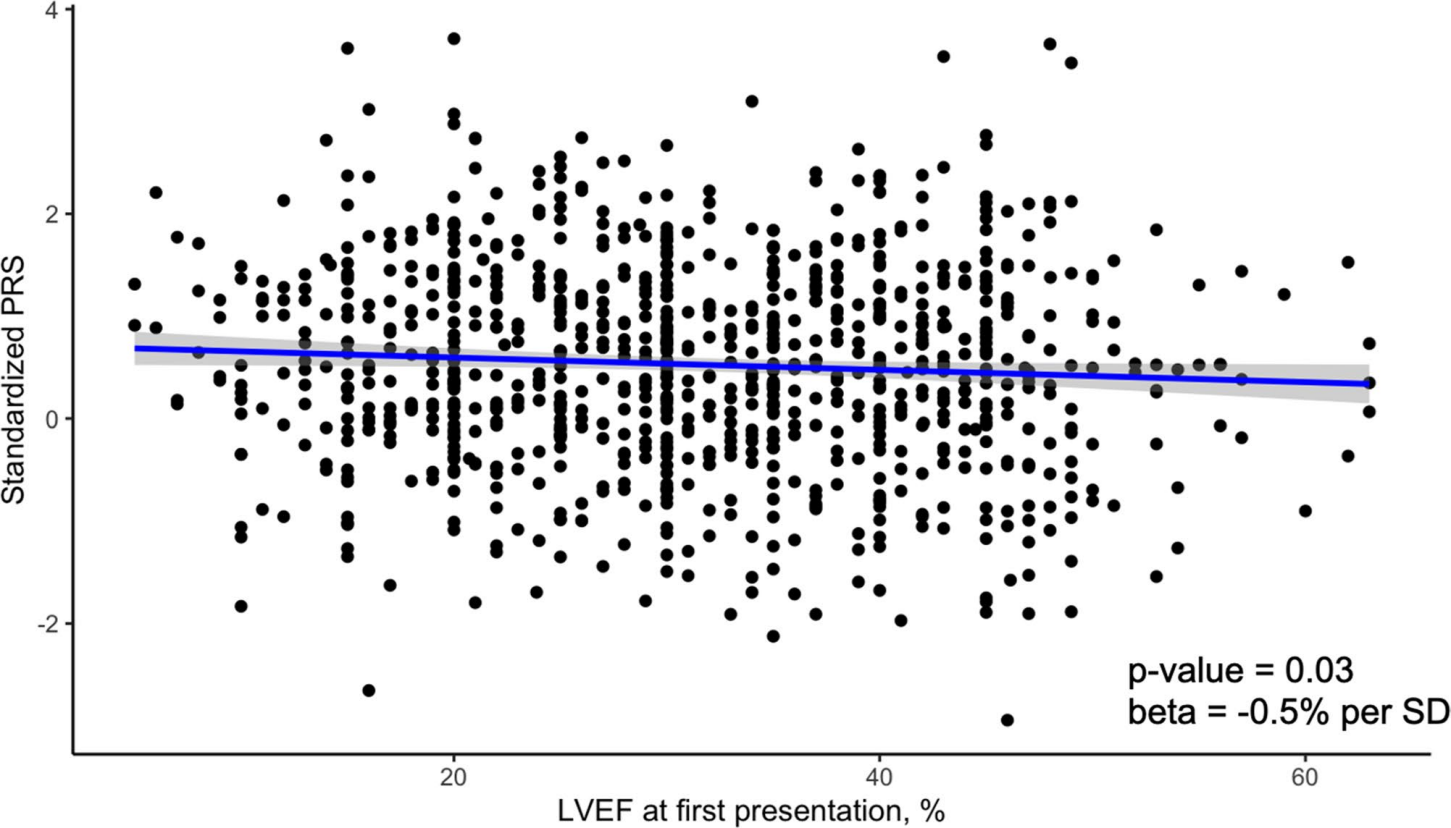
PGS and LVEF at first presentation

PGS demonstrated a signiﬁcant enrichment among DCM cases compared to controls (OR per SD 1.93, *P* = 9.47E-68). This association persisted with consistent edect estimates - ranging from OR 1.5 to 2.2 - across all examined subgroups and demonstrated sudicient statistical signiﬁcance (all *P*<1.58E-06) (Figure 1). Genotype-negative cases had a signiﬁcantly higher PGS compared to genotype-positive individuals (*P* = 0.0015), although PGS was signiﬁcantly enriched in both groups as compared to controls. Furthermore, among DCM cases, higher PGS was signiﬁcantly associated with lower LVEF at ଁrst presentation (*P*=0.03, beta=-0.5% per SD) (Figure 2). There was no signiﬁcant association found between PGS and family history.

**Conclusions**

PGS is strongly associated with risk of DCM, and may modulate the severity of LV dysfunction among DCM cases. Polygenic burden contributes to DCM risk in both genotype-positive and negative cases, although the contribution is stronger in patients without known causative rare variants. Overall, our results suggest a potential clinical applicability for PGS in DCM risk stratiﬁcation.

## A24: Supporting the development of pediatric and orphan medical devices

Anneliene H. Jonker, PhD^1^

^1^Rare Diseases Therapy Development Researcher, University of Twente, Coordinator of DeCODE and Michelle Battye, ERN eUROGEN Programme Manager and partner of DeCODE consortium

*Orphanet Journal of Rare Diseases 2026,* **21(1):**A24

The journey of each baby, child or young person living with a rare disease or severe or complex condition depends on medical devices for their diagnosis, treatment, and care. The average patient in a hospital has 10-15 medical devices around their hospital bed, which increases exponentially with the severity or complexity of the disease. The development of these technologies, the so-called paediatric and orphan medical devices, is slowly gaining much-needed attention. The recent development of the first guidance for the development of orphan devices has, as such, been a significant step forward in the rare diseases landscape. However, while the importance of paediatric and orphan medical devices has been acknowledged, there is a significant unmet need for paediatric and orphan medical devices, specifically those for babies and small children, and there is a high need to stimulate the development of novel or adapted paediatric and orphan medical devices.

To support paediatric and orphan medical device developers, the DeCODe consortium, co-funded by the European Commission, represents a ground-breaking initiative aimed at catalysing device innovation. This collaborative group, comprising clinicians, researchers, industry experts, and regulatory authorities, develops a pivotal platform for developing safe and effective paediatric and orphan medical devices. It will do so to accelerate the development of novel, innovative paediatric and orphan medical device solutions at all stages of the product lifecycle towards implementation. It will map paediatric and orphan medical device stakeholders and initiatives and develop a critical pathway analysis for the optimal development of novel paediatric orphan medical technologies. In spring 2025, an open call will be held for paediatric medical device stakeholders to apply for support for their use cases. Use cases are meticulously selected based on their medical significance, feasibility, and potential impact. Technical assistance is provided to use cases’ applicants, including guidance on device design, engineering, and regulatory compliance tailored to the paediatric population. By uniting the diverse expertise of the consortium, DeCODe aims to facilitate the innovation of developers and surmount regulatory challenges. Ultimately, DeCODe aims to accelerate the development of essential medical devices that enhance and transform the quality of care for our children living with rare diseases.

## A25: Telemedicine applied for at home exercise in a patient with glycogenosis type II

M. Bon^1, 5^, A. Scapin^1,5^, P. Piovani^1,5^, E. Bizzarini^2^, C. Pinzini^3^, L. Verriello^4^, A. Bordugo^1, 5^, M. Scarpa^1, 5^, A. Sechi^1, 5^

^1^Regional Coordinating Center for Rare Diseases, University Hospital of Udine; ^2^SOC MFR - Spinal Unit, Department of Rehabilitation Medicine, IMFR Gervasutta, University Hospital of Udine; ^3^University of Udine - Department of Medical Area - Degree Course in Physiotherapy; ^4^Neurology Unit, University Hospital of Udine; ^5^MetabERN

*Orphanet Journal of Rare Diseases 2026,* **21(1):**A25

**Introduction**

Late-onset Pompe disease (LOPD), is an inherited disorder with autosomal recessive transmission. It is characterized by lysosomal accumulation of glycogen in muscle tissue leading to progressive hyposthenia of the skeletal and respiratory muscles.

LOPD patients require consistent exercise to preserve motor function, but high dropout rates due to boredom are common.

We therefore decided to evaluate the feasibility and effects of a home based exercise protocol, for 6 weeks by a LOPD patient before starting enzyme replacement therapy (ERT).

**Patient and methods**

A 40-year-old female patient diagnosed with LOPD, followed by the Regional Coordination Centre for Rare Diseases in Udine in collaboration with the Gervasutta Hospital, performed a telerehabilitation protocol of 12 personalised exercises at home for 6 weeks using the Rehability^®^Neuro medical device (Imaginary srl). The assesments at t0 (basal) and t1 (after6 weeks) included: two questionnaires, the dynamometric and stabilometric test and the 6-minute-walking test (6MWT).

**Results**

After 6 weeks with Rehability^®^Neuro (Imaginary srl) we noticed an increase in the strength of the intra-rotator muscles of the right shoulder and the extra-rotator muscles of the shoulder bilaterally. Although we did not record any changes in lower body strenght, the data from the 6MWT showed significant distance improvement (from 1980 m to 2772 m) in a self-selected increase speed (from 0.55 m/sec to 0.77 m/sec), with a reduced sense of fatigue and a decrease in heart rate during exercise (from 110 to 104 bpm).

High compliance and patient acceptance were also noted.

**Conclusion**

The results seem to indicate that Rehability^®^ Neuro (Imaginary SRL), appears beneficial for LOPD patients, enhancing motivation and compliance to perform exercises at home. The advantage over traditional systems is to increase patient compliance and motivation.

Further studies on larger cohorts are warranted to assess efficacy and safety of the system.

Consent to publish had been obtained from the patient.

## A26: ERN ReCONNET Red Flags for primary care: an approach to promote early diagnosis in rare and complex conditions

R. Talarico^1,2^, I. Galetti^3^, E. Della Torre^4^, T. Alexander^5^, Jose Ballarin^6^, A. Meyer^7^, I. E. Lundberg^8^, O. Drapalova^9^, S. Schlüter^10^, D. Marinello^1^, M. Cutolo^11^, M. Mosca^1, 2^

^1^Rheumatology Department, Azienda Ospedaliero Universitaria Pisana, University of Pisa (Pisa, Italy), ERN ReCONNET Full Member and Coordinating Healthcare Provider; ^2^Department of Clinical and Experimental Medicine, Rheumatology Unit, University of Pisa, Pisa, Italy; ^3^GILS (Gruppo Italiano Lotta alla Sclerodermia), 50134 Milan, Italy; FESCA (Federation of European Scleroderma Associations), 7500 Saint Maur, Belgium ERN-ReCONNET Patient Advocacy Group; ^4^Università Vita-Salute San Raffaele (Milan, Italy)

Unit of Immunology, Rheumatology, Allergy and Rare Diseases (UnIRAR), IRCCS San Raffaele Scientific Institute (Milan, Italy), ERN ReCONNET Full Member; ^5^Department of Rheumatology and Clinical Immunology, Charité-Universitätsmedizin Berlin, corporate member of Freie Universität Berlin, Humboldt-Universität zu Berlin, and the Berlin Institute of Health (BIH), Berlin, and Deutsches Rheuma-Forschungszentrum (DRFZ Berlin) - a Leibniz Institute, Autoimmunology Group (Berlin, Germany), ERN ReCONNET Full Member; ^6^ERN-ReCONNET Patient Advocacy Group; ^7^Centre de Référence des Maladies Auto-immunes Systémiques Rares, University Hospital of Strasbourg, Strasbourg, France, ERN ReCONNET Full Member. Service de Rhumatologie, University hospital of Strasbourg, Strasbourg, France, ERN ReCONNET Full Member; ^8^Division of Rheumatology, Department of Medicine, Solna, Karolinska Institutet, Stockholm, Sweden, ERN ReCONNET Full Member; ^9^Czech Myositis Working Group, Czech League against Rheumatism, Prague, Czech Republic ERN-ReCONNET Patient Advocacy Group; ^10^Myositis Gruppe, Deutsche Gesellschaft für Muskelkranke e.V., Freiburg, Germany; ERN-ReCONNET Patient Advocacy Group; ^11^Laboratory of Experimental Rheumatology and Academic Division of Clinical Rheumatology, Department of Internal Medicine, University of Genova, 16,132 Genoa, Italy; University of Genova, 16,132 Genoa, Italy; IRCCS Ospedale Policlinico San Martino, Largo Rosanna Benzi, Genoa, Italy, ERN ReCONNET Full Member

*Orphanet Journal of Rare Diseases 2026,* **21(1):**A26

**Abstract**

**Introduction**

The identification of Red Flags for primary care can be crucial as primary care physicians are the first point of contact for people’s healthcare problems in all countries and they can play an important role in shortening and correctly address the pathway for the diagnosis of rare and complex diseases. Therefore, since promotion of early diagnosis is one of the mandate and mission of ERNs, ERN ReCONNET developed an approach for the development of Red Flags in rare and complex connective tissue diseases (rCTDs).

**Material/patients and methods**

The methodological approach foresees 4 phases. The first phase is related to the definition of a Task Force of experts from the ERN ReCONNET Disease Group that guides the development of the Red Flags and that includes methodologists, expert clinicians, expert patient representatives and primary care doctors. In the second phase, a systematic literature review (SLR) is performed to identify signs and symptoms that should raise suspicion of the disease in a primary care setting. During the third phase, a meeting is organised within the Task Force in order to discuss the results of the SLR and prepare the fourth phase. In phase four, a “level of agreement” (LoA) exercise is performed to define a core set of red flags based on an agreement of ≥ 95% on consideration as red flag among experts and on a LoA ≥ 8 on a 0 to 10 points scale. The LoA is performed by means of a dedicated online tool. After the Red Flags have been identified a scientific paper is published by members of the Task Force and a dedicated dissemination campaign is launched by ERN ReCONNET.

**Results**

The methodological approach was already applied in the ERN ReCONNET IgG4-RD Disease Group, 9 red flags were identified, from which five reached a 100% agreement. The paper was already submitted and accepted for publication. Currently, the ERN ReCONNET Disease Group on Inflammatory Myopathies is also applying the methodology and other Disease Groups withing the Network will follow.

**Conclusion**

The identification of Red Flags for the early recognition of rare and complex diseases specifically intended for use in primary care within ERN ReCONNET represents a valuable instrument for the early referral to tertiary care centres to better define the diagnosis. Therefore, the development of Red Flags can have a great impact on promoting earlier diagnosis and reduce clinical, psychosocial, and economic burdens for patients and challenges for healthcare systems, shortening the time between the onset of symptoms and disease recognition is key to optimizing the treatment of complex diseases.

## A27: “See My Life” - AN international, multidisciplinary research project aimed at evaluating the quality of life of children with visual impairment caused by rare eye diseases

Katarzyna Nowomiejska^1^, Caroline Wernert-Iberg^2^, Bernard Coupez^2^, Isabella Vacchi^2^, Valentine Gourinat^4^, Hélène Dollfus^2,3^

^1^Medical University of Lublin, Lublin, Poland; ^2^ERN-EYE Coordination Team, Hôpitaux Universitaires de Strasbourg, Strasbourg, France; ^3^CARGO, Hôpitaux Universitaires de Strasbourg, Strasbourg, France; ^4^Strasbourg University, Strasbourg, France

*Orphanet Journal of Rare Diseases 2026,* **21(1):**A27

**Introduction**

In Europe, rare eye diseases (RED) are a leading cause of severe visual impairment (VI) or blindness in children. These conditions not only result in significant physical challenges but also lead to psychological distress and social hindrance, greatly affecting the quality of life for both the affected children and their families. While the physical difficulties are already well understood, the psychological, emotional, relational, and social impacts of RED and VI are often underrepresented and not adequately measured.

**Objectives**

“See My Life” aims to identify, quantify, and analyse these challenges to better assess care and treatment strategies for children with RED. The study is conducted in six European countries (Germany, Belgium, France, Italy, Lithuania, and Poland) by multidisciplinary teams including medical professionals, social scientists, and psychologists. Each team works with over 100 children and adolescents to assess the various aspects of life affected by their vision loss, such as social relationships, daily habits, education, and emotional well-being. The study will also involve parents, caregivers, and healthcare providers to gather a comprehensive understanding of the impacts on the children’s environments.

**Material and method**

Each participant undergoes three clinical visits, where visual assessments will be conducted alongside with validated questionnaires focused on their life experiences and the challenges they face due to VI. A subset of participants also participates in psychological interviews to provide more detailed insights into their daily lives and emotional experiences. Family members and caregivers are invited to participate in similar interviews to help deepen the understanding of children’s lived experiences.

**Results**

At this stage, we have enrolled 176 patients and completed 119 interviews out of the 600-924 patients and 180-270 interviews planned. While data collection and analysis are still ongoing, results so far are promising, with key themes and outcomes already emerging, suggesting potential future applications in specialized institutions and healthcare teams. Preliminary results from the interviews already reveal several noteworthy areas, such as family organisation (parental over-protection may be a constraint on the development of the child’s autonomy), daily social difficulties (VI children with disorders associated with their ADR have more difficulty socialising), leisure activities (early participation in sports is highly beneficial for both quality of life and motor skill development), or the specific constraints associated with assistive technologies (high price, long time to obtain, stigmatisation caused by identifying devices), etc.

**Conclusion**

The research aims to develop tools to assess the quality of life in children and adolescents with severe visual impairment due to RED. These tools will improve the care provided to young patients by better aligning healthcare services with their actual needs. The findings are expected to serve as a reference across Europe and globally, benefiting a broad range of individuals affected by similar conditions.

## A28: Small Scale Radiomics in Neuroimaging: Potential as a Clinical Support Tool

Júlia Romagosa^1^*, Arnau Valls-Esteve^1,2^, Christian Mata^1,4^, Sandra Bernaus^1^, Jordi Muchart^1,3^, Emilio Inarejos^3^, Àngels Garcia-Cazorla^5,6^, Christian Stephan-Otto^1,7^

^1^Pediatric Computational Imaging Center, Institut de Recerca Sant Joan de Déu, Esplugues de Llobregat, Spain; ^2^Innovation Department, Hospital Sant Joan de Déu, Esplugues del Llobregat, Spain; ^3^Diagnostic Imaging Department, Hospital Sant Joan de Déu, Esplugues del Llobregat, Spain; ^4^Automatic Control Department, Universitat Politècnica de Catalunya, Barcelona, Spain; ^5^Neurology Department, Neurometabolic Unit, Hospital Sant Joan de Deu, Esplugues de Llobregat; ^6^Centro de Investigación Biomédica en Red de Enfermedades Raras (CIBERER), Madrid, Spain; ^7^Centro de Investigación Biomédica en Red de Salud Mental (CIBERSAM), Madrid, Spain

*Correspondence: Júlia Romagosa (julia.romagosa@sjd.es)

*Orphanet Journal of Rare Diseases 2026,* **21(1):**A28

**Introduction**

Conventional neuroimaging techniques have limited utility in diagnosing some rare neurological diseases, as they often fail to capture the subtle structural abnormalities characteristic of these conditions. Nevertheless, advanced neuroimaging analysis techniques are more precise in quantitative evaluation and have the potential to identify novel biomarkers that can aid in both diagnosis and prognosis. This study aims to describe cerebral volumetric alterations in a cohort of Spanish patients diagnosed with neurometabolic disorders, a subset of rare neurological diseases, using advanced neuroimaging techniques.

**Materials/Patients**

Our institution, a reference center for rare neurological diseases, is part of several consortia dedicated to advancing the understanding and treatment of these conditions, such as MetabERN, dedicated to hereditary metabolic disorders, and ERN-ERD, dedicated to neurological diseases. High-resolution neuroimaging data are continuously collected for clinical and research purposes, for example from patients diagnosed with STXBP1, SYNGAP1 and GRIN neurodevelopmental disorders.

**Methods**

We developed and implemented an advanced neuroimaging pipeline designed to achieve precise segmentation of key brain structures. Each segmented region was assigned a volumetric score with respect to the individual’s brain volume and compared against a database of sex- and age-matched healthy control subjects. These volumetric assessments allowed us to detect differences between patient groups based on clinical diagnosis, subgroup classifications, and their correlation with specific neurological symptoms. Statistical analyses were employed to evaluate these relationships and to identify characteristic patterns of brain volume deficits.

**Results**

Previous research conducted by our group with a small cohort of neurotransmitter disorders has indicated that distinct volumetric deficits can be associated with different clinical symptoms, regardless of the overarching clinical diagnosis. Furthermore, preliminary analyses with the neurometabolic disorders cohort proposed here have revealed specific volumetric deficits in certain brain regions according to their clinical presentation. For example, alterations in the STXBP1 gene, a neurodevelopmental disorder often causing neurocognitive and motor dysfunction, present a distinctive cerebellar volume deficit pattern.

**Conclusions**

Our findings underscore the potential of advanced neuroimaging analysis techniques as a valuable tool for identifying diagnostic and prognostic biomarkers in rare neurological diseases, particularly in neurometabolic disorders. The distinct volumetric patterns observed in this patient cohort could provide crucial insights into disease mechanisms and symptomatology. Nonetheless, further research is necessary to validate these preliminary results and fully explore their potential for clinical application in personalized diagnosis and treatment planning for rare diseases.

## A29: The genetic landscape of dilated cardiomyopathy in Hungary

Beáta Csányi^1^, Lidia Hategan^1^, János Borbás^1^, Viktória Nagy^1^, Hedvig Takács^1^, Attila Pálinkás^2^, Eszter Dalma Pálinkás^1^, Miklós Rábai^3^, László Balogh^4^, Róbert Halmosi^3^, Attila Borbély^4^, Tamás Habon^3^, Noémi Nyolczas^5^, István Nagy^6^, Zoltán Hegedűs^7,8^, Róbert Sepp^1^

^1^Department of Internal Medicine and Cardiology Center, University of Szeged, Hungary; ^2^Erzsébet Hospital, Hódmezővásárhely, Hungary; ^3^Department of Internal Medicine and Cardiology Center, University of Pécs, Hungary; ^4^Cardiology Center, University of Debrecen, Hungary; ^5^Gottsegen György” National Institute of Cardiology, Budapest, Hungary; ^6^Seqomics Biotechnology Ltd., Mórahalom, Hungary; ^7^Institute of Biophysics, Biological Research Centre, Szeged, Hungary; ^8^Department of Biochemistry and Medical Chemistry, University of Pécs, Pécs, Hungary

*Orphanet Journal of Rare Diseases 2026,* **21(1):**A29

**Keywords:** *Dilated cardiomyopathy, Mutation, Next-generation sequencing*

**Background**

Dilated cardiomyopathy (DCM) is a heart muscle disease characterized by dilation and decreased systolic function of the left or both ventricles. In at least 25% of cases, familial accumulation can be detected, with autosomal dominant inheritance. The latter cases are caused by mutations in cytoskeletal, sarcomeric/Z-band, nuclear membrane or intercalary discus coding genes, of which the involvement of the titin gene is the most common, according to literature data.

**Objective**

In our study, we aimed to determine the mutation spectrum of the Hungarian DCM patient population by sequencing a large number of Hungarian DCM patients.

**Betegek és módszerek**: One hundred and thirty-five patients with dilated cardiomyopathy (86 men, 49 women, average age 52±16 years) were genetically examined. Genetic analysis was performed by next-generation sequencing, validation was performed by capillary sequencing. During next-generation sequencing, a total of 98 genes known to cause cardiomyopathy were resequenced in a total of 98 targeted regions, with 500,000 base pairs covered by the sequenced target region.

**Results**

During genetic testing, 32 patients (24%) were found to have a pathogenic (P) or possibly pathogenic (LP) genetic variant. Two patients (1.5%) had a double P/LP variant. The most commonly affected P/LP variants were the titin gene (TTN, 54%), the beta myosin heavy chain gene (MYH7, 17%), and the desmoplakin gene (DSP, 12%). Other genes affected by P/LP variants were troponin T (TNNT2), RNA binding motif protein 20 (RBM20), BAG cochaperone 3 (BAG3), phospholamin (PLN), and lamin A/C genes (LMNA). The vast majority of the P/LP TTN and DSP variants were truncating variants (stop codon or frame-shift), while the P/LP variants of the MYH7 gene were predominantly point mutations. Among non-P/LP carriers, 54 patients (40%) carried a ‘variant of unknown significance’ (VUS). The genes most commonly affected by VUS were TTN (41%), DSP (15%) and MYH7 (11%).

**Conclusion**

Our results suggest that genetic variants that can be considered pathological can be detected in about 25% of the Hungarian DCM patient population. The latter variants most often affect the TTN gene. The above mutation spectrum is similar to data from other European DCM patient populations.

## A30: The development of an undiagnosed respiratory patient pathway – clinical procedures and the patient perspective in the field of rare respiratory diseases

European Reference Network for Rare Diseases of the Respiratory System (ERN-LUNG), University Hospital Frankfurt.

This paper is written on behalf of the “UnDiagnosed Patients Working Group” from ERN-LUNG.

Lisa Rist^1^, Thomas Wagner^2^, Olivia Steinmann^3^, Andrea Cembrero Bonet^4^, Matt Bolz-Johnson^5^, Gergely Meszaros^6^, Elisabetta Balestro^7^, Anna Kerpel-Fronius^8^, Veronika Müller^9^, Katarzyna Rososinska^10^, Irene Lang^11^, Petra Pennekamp^12^, Peter Dorfmüller^13^, Holger Storf^14^, Alexandra Berger^15^

^1^Frankfurt Reference Centre for Rare Diseases, University Hospital Frankfurt, Goethe University, Frankfurt, Germany; ^2^Frankfurt Reference Centre for Rare Diseases, University Hospital Frankfurt and ERN-LUNG, Frankfurt, Germany; ^3^Frankfurt Reference Centre for Rare Diseases, University Hospital Frankfurt, Goethe University, Frankfurt, Germany; ^4^EURORDIS – Rare Diseases Europe, Paris, France; ^5^EURORDIS – Rare Diseases Europe, Paris, France; ^6^University Hospital Frankfurt, Frankfurt, Germany; ^7^University Hospital of Padua, Padua, Italy; ^8^National Korányi Institute for Pulmonology, Budapest, Hungary; ^9^Semmelweis, Budapest, University, Hungary; ^10^Saarland University Medical Center, Homburg, Homburg, Germany; ^11^Medical University of Vienna, Vienna, Austria; ^12^University Hospital Muenster, Muenster, Germany;^13^University Hospital of Giessen and Marburg and Institute for Lung Health Giessen, Giessen, Germany; ^14^University Hospital Frankfurt, Goethe University, Frankfurt, Germany; ^15^Frankfurt Reference Centre for Rare Diseases, University Hospital Frankfurt, Goethe University, Frankfurt, Germany

**Correspondence:** Lisa Rist (gietz@med.uni-frankfurt.de)

*Orphanet Journal of Rare Diseases 2026,* **21(1):**A30

**Keywords**: *European Reference Network for Rare Diseases of the Respiratory System (ERN-LUNG); Undiagnosed Patient Pathway; Misdiagnosed patient pathway; patient advocates; diagnostic pathway; patient journey*

**Abstract**

**Introduction**

This paper describes the methodology of an undiagnosed patient pathway (UDPP), a structured approach to improve the diagnostic and therapeutic pathway for patients with complex respiratory problems, making sure that only those are referred to the ERN who need this expert service and excluding those with more common and easy to diagnose rare diseases of the lung. The UnDiagnosed Patients Working Group (UDP-WG), consisting of patients, patient organizations, clinicians, and researchers was established within the European Reference Network for Rare Diseases of the Respiratory System (ERN-LUNG) to develop a standard operating procedure (SOP) for referral of undiagnosed patients to ERN-LUNG. Undiagnosed in this paper refers both to patients whose conditions have not been identified and those which have been misdiagnosed.

**Methods**

Three workstreams - patient, clinical, and research - were formed to develop the UDPP. Through patient workshops insights were gained into patient journeys, with the focus on needs, barriers, and enablers for diagnosis and patient empowerment. The clinical workstream, organized in task force groups, identified key diagnostic procedures for more common rare diseases like cystic fibrosis (CF), interstitial lung diseases (ILD), primary ciliary dyskinesia (PCD), and pulmonary hypertension (PH). The resulting UDPP, is then translated into a SOP by testing the clinical checklist on retrospective patient cases.

**Results**

Coordination of multidisciplinary care is crucial for timely diagnosis of multi-system rare conditions. By defining key diagnostic procedures for CF, ILD, PCD, and PH compiled into a checklist and excluding those patients from referral, this process helps to identify patients with undiagnosed respiratory symptoms which should be referred to the ERN-LUNG system for further evaluation. Patient empowerment is the most important aim within the patient workstream of the UDP-WG and is supported by the formulation of recommendations, which were analysed based on the patients’ experiences and insights focusing on needs, barriers and enablers. The procedure proposed can streamline referrals to specialists to reduce diagnostic delays, misdiagnosis, inefficient utilization of resources, and ineffective and/or harmful treatments.

**Conclusion**

The UDPP consists of the clinical checklist and the recommendations from the patient perspective. It provides a framework for primary and specialist healthcare services and a guide for navigation through the healthcare system for the patients, their families as well as healthcare workers. The UDPP development is an innovative approach to streamline the patient journey. This approach can be utilized by other ERNs for their respiratory undiagnosed patient programs. Pilot implementations of the UDPP can further demonstrate its potential to streamline the diagnostic journey, offering a model for healthcare systems to better serve the undiagnosed.

## A31: Let your ERN patient registry make more FAIR impact using MOLGENIS EMX2

G. Been^1^, J. van der Velde^1^, E. van Enckevort^1^, E. Hyde^1^, The MOLGENIS Team, M. Swertz^1^

^1^Department of Genetics, University of Groningen, University Medical Center Groningen, HPC CB50, P.O. Box 30001, Groningen, 9700 RB, The Netherlands

*Orphanet Journal of Rare Diseases 2026,* **21(1):**A31

**Background/Objectives**

Your data in the life sciences can have far more impact when it is ﬁndable and (re)usable by a larger audience. In the past years MOLGENIS EMX2 has been implemented in registries for multiple ERNs such as ERN-GENTURIS, ERN-ITHACA, ERN-SKIN, ERN-CRANIO and ERN-ReCONNET. Facilitating their data collection, support research, enhance collaboration and to promote best practices.

**Methods**

To achieve this, we have developed MOLGENIS EMX2, the 5th generation of the MOLGENIS open source scientiﬁc data platform with 100+ active installations powering a variety of life science web applications including patient mutation registries, catalogues, research portals and omics data. Importing and exporting of data is easy and achieved through Excel and CSV ﬁles. Advanced users adopt our dedicated Python client, export RDF, use GraphQL, or R. For instance, this allows data managers to easily transfer data from their local environment into an ERN patient registry.

MOLGENIS EMX2 facilitates automated data extraction, as well as manual uploads.

**Results**

For users, we have developed attractive data displays, dashboards, query screens, data entry forms and tools to manage fully ﬂexible data structures and user permissions. For machines, interfaces such as FAIR Data Point and Beacon v2 can be registered at search portals to make the contents of these applications known to the wider world such as ERDRI and ERDERA. Interoperability is driven by pre-deﬁned best-practice semantic datamodel templates for biobank-, cohort- and harmonisation-catalogues, FAIR Genomes, JRC Common Data Elements, DCAT, Beacon v2, SolveRD-RD3, all based on popular resources including MIABIS, NCIT, HPO, ORDO and DUO. Datamodels are easily extended, e.g. there is a metabolomics add-on for X-Omics.

**Conclusion**

We are delighted to present EMX2, the next generation of the MOLGENIS FAIR data platform. MOLGENIS EMX2 is open-source (GNU LGPL v3.0) and available at *https://github.com/molgenis/molgenis-emx2*. Install it yourself or contact us for hosting options.

**MOLGENIS EMX2 is supported by** ERNs GENTURIS, ITHACA, Skin, ReCONNET and CRANIO and other projects such as Solve-RD, EJP-RD, EOSC4CANCER, BBMRI, LifeCycle, ATHLETE, LongITools, ConcePTION, IHEN, STAGE.

## A32: Role of the clinical research coordinator within international networks for congenital heart disease

Laura Schianchi^1,*^, Anna Rietkoetter^2^, Chiara Corrado^3^, Nathasha Samali Udugampolage^4^, Gerhard-Paul Diller^2,5,6^ and Massimo Chessa^1,6,7^

^1^Department of Pediatric and Adult Congenital Heart Disease, IRCCS Policlinico San Donato, San Donato Milanese (Milan), Italy; ^2^Department of Cardiology III-Adult Congenital and Valvular Heart Disease, University Hospital Muenster, Muenster, Germany; ^3^Department of Congenital Cardiac Surgery, IRCCS Policlinico San Donato, San Donato Milanese (Milan), Italy; ^4^Cardiovascular-Genetic Centre, IRCCS Policlinico San Donato, San Donato Milanese (Milan), Italy; ^5^Adult Congenital Heart Centre and National Centre for Pulmonary Hypertension, Royal Brompton and Harefield National Health Service Foundation Trust, Imperial College London, London, UK; ^6^European Reference Network for Rare, Low Prevalence and Complex Diseases of the Heart-ERN GUARD-Heart; ^7^Vita-Salute San RaYaele University, Milan, Italy

*Correspondence: Laura Schianchi, (laura.schianchi@grupposandonato.it)

*Orphanet Journal of Rare Diseases 2026,* **21(1):**A32

**Background**

Conducting high-quality research in congenital heart disease (CHD) poses unique challenges due to its low prevalence, variability in clinical presentations and treatments, and the complexities of involving pediatric patients in clinical trials. While multicenter collaborations, enabled by international research networks, are essential for larger studies that enhance understanding and standardize CHD practices globally, doctors often do not have the time to manage the many coordinating and organizational tasks required. This is why the role of clinical research coordinators (CRCs) has become increasingly important. CRCs act as the crucial link between doctors and trial participants, managing logistics, overseeing clinical data, and maintaining communication with patients needing long-term follow-up. Despite their growing importance, the number of CRCs remains limited, and there is a pressing need for more professionals and specialized training programs to fill this gap. This study seeks to delineate the role of CRCs in CHD research, and the speciﬁc skill set and competencies required to fulfil this role effectively.

**Materials and methods**

This project represents a transformative initiative designed to improve the quality and efficiency of CHD research by advancing a deeper understanding of the role of clinical research coordinators (CRCs). This effort aims to increase the number of CRCs, establish a dedicated network, and create specialized training programs. First, a state-of-the-art review will be conducted, providing a comprehensive and cutting-edge synthesis of recent advancements in international CHD research collaborations, with a focus on the evolving roles, skills, and competencies of CRCs. Following this, we will develop a rigorously structured questionnaire to be distributed across leading CHD centers within the European Reference Network (ERN). This survey will evaluate the presence of dedicated CHD CRCs and examine their professional backgrounds and work experiences. Finally, the ﬁndings will inform the establishment of a CRC network for regular meetings and the development of specialized training programs tailored to the needs of CHD CRCs, ultimately enhancing future research collaborations and setting new standards of excellence in CHD clinical research.

**Conclusions**

In conclusion, this project will provide critical insights into the evolving role of CRCs in CHD research, addressing a key gap in the current understanding of their skills and competencies. By mapping the presence and expertise of CHD CRCs across leading European centers, the ﬁndings will not only enhance research coordination but also drive the development of specialized training programs. These initiatives will ultimately improve the quality of multicenter collaborations, fostering more efficient and effective CHD research internationally.

## A33: Thyroid hormone analogue (TRIAC) therapy for resistance to thyroid hormone in children: proposal for an international multi-center clinical trial

W. Edward Visser^1^

^1^Rotterdam, The Netherlands, Endo-ERN (chair of Thyroid)

*Orphanet Journal of Rare Diseases 2026,* **21(1):**A33

**Introduction**

Thyroid hormone is crucial for normal development and metabolism of virtually all tissues. The genomic effects of thyroid hormone are exerted through binding of T3 to its nuclear receptor (TR), which functions as a ligand-dependent transcription factor. TRα is expressed in heart, bone, gut and brain, and TRβ in liver, kidney and pituitary. Mutations in TRβ cause Resistance to Thyroid Hormone β (RTHβ) (~1/40,000 individuals) with TRβ-expressing tissues being refractory to thyroid hormone while TRα-expressing retain normal sensitivity. RTHβ displays a biochemical hallmark of elevated thyroid hormones with non-suppressed TSH concentrations and a wide range of disease features starting in childhood including goiter (thyroid), AHDS and learning difficulties (brain), tachycardia and heart failure (heart).

**Knowledge gap & preliminary observations**

There is a large unmet clinical need as no registered treatment is available. Effective treatment of RTHβ should normalize the over-stimulation of TRα-expressing tissues while avoiding under-stimulation of the TRβ-expressing tissues. The T3 analogue Triac has preferential affinity for TRβ in the treatment of RTHβ. We showed positive effects of Triac in all types of TRβ mutations in in vitro studies along with beneficial effects in patients in a retrospective multi-center study (unpublished). No clinical trial has investigated the effectiveness of Triac in RTHβ patients.

Previously, we led an investigator-initiated international (including Endo-ERN members) clinical phase 2 trial (Triac Trial I), showing effectiveness and safety of Triac in another rare thyroid disorder (MCT8 deficiency), providing the basis for a submission to the EMA (2023).

**Proposal**

We propose to conduct an investigator-initiated international clinical phase 2 trial to investigate the efficacy of Triac in paediatric patients with RTHβ with the explicit aim to advance this orphan drug towards approval by regulatory authorities expanding the expected label for MCT8 deficiency.

As **study design** we propose to perform a trial in 20 paediatric, genetically proven, RTHβ patients (based on power calculation). The comparator arm is under discussion.

**Intervention**: Patients will be treated with Triac for 24 months; dose escalation will be informed by our previous Triac Trial I.

**Outcomes**: Primary outcome is a reduction of FT3 and FT4 concentrations into the reference interval. Secondary outcomes are reduction of tachycardia (using 24 h Holter analysis) and of anxiety/ADHD symptoms.

Exploratory outcomes are improvement of bone mineral density, bone age, normalization of energy expenditure. Other outcomes are improvement of stool frequency and diarrhea, reduced perspiration and improvement of body weight. Safety assessments include questionnaires and biochemical tests.

Extensive clinical, biochemical, neurocognitive and cardiac phenotyping will be performed at baseline, after 6, 12, 18 and 24 months.

**Conclusion**

The conduct of an international multi-center clinical trial is crucial to advance Triac as a promising therapy to address the large clinical unmet need in RTHβ.

## A34: Prehabilitative training in children and adolescents with soft tissue or bone sarcoma of the lower extremity – protocol of a feasibility study

Queisser Jennifer^1, 2^, Schmid Irene^3^, Oberhoffer-Fritz Renate^2^, Wörtler Klaus^4^, von Luettichau Irene1, Kesting Sabine^1, 2^

^1^Department of Pediatric Hematology, Oncology, and Stem Cell Transplantation, Children’s Cancer Research Center, Kinderklinik München Schwabing, TUM School of Medicine and Health, TUM University Hospital, Klinikum rechts der Isar, Technical University of Munich, 80804 Munich, Germany; Children’s Oncology Network Bavaria, KioNet, 91054 Erlangen, Germany; ERN Member; ^2^Chair of Preventive Pediatrics, Department Health and Sport Sciences, TUM School of Medicine and Health, Technical University of Munich, 80992 Munich, Germany; ^3^Department of Pediatric Oncology and Hematology, Dr. von Haunersches Kinderspital, Ludwig-Maximilians-University Munich, 80,337 Munich, Germany; Children’s Oncology Network Bavaria, KioNet, 91054 Erlangen, Germany; ERN Member; ^4^Section of musculoskeletal Radiology, Department Clinical Medicine, TUM School of Medicine and Health, TUM University Hospital, Klinikum rechts der Isar, Technical University of Munich, 81675 Munich, Germany

*Orphanet Journal of Rare Diseases 2026,* **21(1):**A34

**Introduction**

Soft tissue and bone sarcomas of the lower extremity pose significant challenges for affected individuals, often associated with considerable burden. Chemotherapy, load restrictions, and surgery frequently result in long-term physical limitations, causing structural and functional deterioration. In childhood and adolescence, these challenges are particularly pronounced, as they affect physiological development, resilience, and autonomy. Although movement promotion and therapeutic programs are designed to address these deficits, they are typically implemented post-operatively and during follow-up care. The benefit of implementing a specific program before the operative therapy remains elusive. In the presented study we propose to explore the feasibility of a supervised prehabilitative training intervention and gather preliminary data on its potential effects in enhancing the pre-operative condition. The goal is to improve post-operative outcome and rehabilitation in children and adolescents diagnosed with soft tissue or bone sarcoma of the lower extremity.

**Material/Patients and Methods**

This bicentric feasibility study, designed as a controlled clinical trial, enrolls all children and adolescents aged 6-18 years who are newly diagnosed with primary osteosarcoma, Ewing’s sarcoma, or rhabdomyosarcoma of the lower extremity. Based on the study site, participants are allocated to either the intervention group (IG) or control group (CG), with a target sample size of 16–18. The intervention consists of specific strength and balance training sessions during neoadjuvant therapy, conducted at least twice a week for a minimum of 30 min per session. The CG does not receive any training intervention. The study has been consented by the local ethics committee and it will be registered on ClinicalTrials.gov before recruitment starts.

**Endpoints/Results**

The primary endpoint is the proof of feasibility of the intervention, assessed via descriptive analysis of recruitment rate, acceptance, data quality, practicability, and safety (adverse events). The secondary endpoint is the demonstration of the efficacy of the intervention comparing structural and functional measurements intra-individually and between groups at four timepoints: within ten days post-diagnosis, pre-operatively (post-intervention), at the end of therapy, and at 1-year follow-up. The measurements include psoas muscle area, body composition, strength, mobility, balance ability, gait analysis, two questionnaires on physical activity and quality of life, and quantitative measures of the clinical course during treatment (days of hospitalization, infection rates, etc.).

**Conclusion**

This study is designed to evaluate the feasibility of a specific prehabilitative training intervention in children and adolescents with soft tissue or bone sarcoma of the lower extremity.

Additionally, we collect preliminary data on the effects of this training, aiming to mitigate muscle mass loss, support physiological body composition, and improve functional outcomes such as balance, gait, and physical activity. Enhancing everyday functionality and fostering a sense of autonomy can significantly improve the quality of life in this population, underscoring the importance of investigating and promoting such interventions in this underrepresented patient group.

## A35: Leveraging FAIR principles to enable collaborative data exploration and analysis by people and machines

Simone Louisse^1,2^, Marco Roos^3^, Daphne Wijnbergen^3^, Nawel Lalout^4-6^, Mark D. Wilkinson^7^, Luiz Bonino^3,8^, Ana Rath^9^

^1^ERN GUARD-Heart, Amsterdam UMC, Amsterdam, The Netherlands; ^2^Hart4Onderzoek (Heart4Research), Haarlem, The Netherlands; ^3^Department of Human Genetics, Leiden University Medical Centre, Leiden, The Netherlands; ^4^ERN EURO-NMD, Paris, France; ^5^Duchenne Parent Project, Veenendaal, The Netherlands; ^6^Department of Rare Diseases, RadboudUMC, Nijmegen, The Netherlands; ^7^Departamento de Biotecnología-Biología Vegetal, ETSI Agronómica, Alimentaria y de Biosistemas, Centro de Biotecnología y Genómica de Plantas (CBGP, UPM-INIA/CSIC), Universidad Politécnica de Madrid, Madrid, Spain; ^8^Services and CyberSecurity group, University of Twente, Enschede, The Netherlands; ^9^Inserm, US14 - Orphanet, Plateforme Maladies Rares, Paris, France

**Correspondence:** Simone Louisse (slouisse62@gmail.com)

*Orphanet Journal of Rare Diseases 2026,* **21(1):**A35

**Abstract**

People and families living with rare conditions encourage the reuse of their data for patient benefit, which implies purposes other than those for which the data were originally collected, and by other users^1^. They expect that patients will benefit greatly when data are by default prepared for running validated state-of-the-art statistical and learning workflows automatically over multiple datasets.

Patient Advocates (PAs) repeatedly confirm the importance of preparing data for such reuse. Aware of progress in data management, they articulate that (i) multiple types of data in multiple locations need to be findable and computationally usable as if they were virtually in one database, while remaining distributed; (ii) all stakeholders (including patients, clinicians, data scientists, regulatory experts, policy makers) need to be empowered to make the best use of distributed data, also implying that stakeholders leverage each others expertise (e.g. data scientists develop automated workflows for questions of patients and clinicians). This requires a substantial multi-stakeholder investment, often without a direct benefit for the original data producers.

The EJP RD initiated the development of a ‘Virtual Platform’ (VP) to enable automated discovery and use of distributed, heterogeneous data. It provides different interfaces for different stakeholders. We explain the concept by a scenario, starting with a PA proposing a rare disease researcher to calculate the time to diagnosis per country as an important indicator. This should be automatically recalculated when more resources connect to the VP and when care pathways change. The PA and the researcher find the VP Portal via Orphanet and an ERN web site, and use the portal’s resource discovery function to explore connected resources (e.g. ERN registries). They reach out to a data scientist for automating the calculation. She finds that VP resources have an ‘Application Programming Interface to explore them via code. She further finds ontology-based, machine-actionable models for resources to describe their access protocols, data use conditions, data catalogues (e.g. what diseases a registry covers), data elements, and relations between data elements (e.g. Patient *diagnosed-with* Diagnosis). The models use Web technology to provide each type and relation a globally unique identifier: the basis for interconnecting the resources comprising the VP network. These are used in the code that calculates the time-to-diagnosis, selecting resources by their ontological descriptions. The PA, researcher and data scientist execute the calculation, discuss the results, and repeat this regularly.

The scenario demonstrates how the VP supports leveraging the expertise of multiple stakeholders. The *FAIR Guiding Principles for scientific data management and stewardship*^2^ guided the design of a network that can function as one system. We demonstrate direct involvement of PAs in instigating interdisciplinary collaboration to address their questions. The focus shifts from downloading data to visiting data resources to answer questions.

**ERN**

ERN GUARD-Heart, ERN EURO-NMD

## A36: Multistakeholder mapping of endocrine medicine availability and shortages in Europe: Endo-ERN expert centres

Johan de Graaf (ePAG)^1,3,5^ , Petra Bruegmann (ePAG) ^2,5,7^, Emily White (Endo-ERN) ^3,4,5^, Dirk de Rijdt (ESE)^6^

^1^Dutch Pituitary Foundation, Nijkerk, The Netherlands; ^2^German Network of Pituitary and Adrenal Diseases, Fürth, Germany; ^3^Department of Endocrinology, Leiden University Medical Center, Leiden, The Netherlands; ^4^Department of Endocrinology and Metabolism, Amsterdam University Medical Center, Amsterdam, The Netherlands; ^5^Endo-ERN European Reference Network on Rare Endocrine Conditions; ^6^European Society of Endocrinology, Bristol, UK; ^7^European MEN Alliance (EMENA), München, Germany

**ERN**: Endo-ERN

**Contact PI:** Johan de Graaf (j.degraaf@hypofyse.nl)

*Orphanet Journal of Rare Diseases 2026,* **21(1):**A36

**Abstract**:

Medicine shortages are a critical issue for physicians and patients in the rare disease community. Medicines and orphan drug shortages or supply issues may have various causes, such as lack of active product ingredients (API), pricing and reimbursement hurdles, withdrawal from market because of commercial or clinical reasons. Such varied causes demand very different solutions and prioritization to increase supply. The impact of drug shortages may be very variable: in some cases similar alternative products are available, in other situations where alternatives do not exist or have a different safety and efficacy profile, there may be an impact on disease control and patient quality of life. For patients reliant on orphan drugs (ODs) as the only available therapy this issue becomes critical. This study is a joint effort between European Society of Endocrinology (ESE), European Society for Paediatric Endocrinology (ESPE) and their respective Patient Advocacy Groups (PAG). The ESE Patient Advocacy Group Board issued a small survey on drug shortages amongst the patient representatives affiliated with the society in spring 2023. These initial results indicated that discrepancies in drug availability are more numerous than originally estimated, signaling that a more extensive study is needed to map the current situation and the variance between different countries. Therefore, we have issued a multi-stakeholder survey targeting three key groups: National Societies of EU countries, Endo-ERN expert centres and patients/patient advocacy groups for rare endocrine conditions. Data collection for the first and second round of this survey have begun via the ESE, ESPE, and Endo-ERN (targeting National Societies and Endo-ERN expert centres respectively). The third round of this survey i.e. patient and patient representatives will be launched by the end of 2024. The final results will be summarized into a report mapping the state and impact of medicine shortages throughout Europe for patients with rare endocrine conditions and proposals of actions to ameliorate these shortages experienced by patients and Heathcare providers alike.

## A37: Accelerating and reducing the cost of literature reviews and regulatory dossiers for rare diseases with AI

M. Lootus^1^, L. Beatson^1^, L. Gold^1^

^1^Tehistark, London, UK

*Orphanet Journal of Rare Diseases 2026,* **21(1):**A37

**Introduction**

Regulatory dossier preparation is a critical but resource-intensive process for research institutions, pharmaceutical and healthcare companies, particularly given the increasing complexity of global regulatory frameworks. This challenge is amplified in the context of rare diseases, where limited patient populations and scarce clinical data add to the complexity. This study demonstrates the application of AI technologies, specifically natural language processing (NLP) and machine learning (ML), to automate the generation of regulatory dossiers. The goal is to streamline the process, enhance accuracy, and reduce the time required for regulatory submissions.

**Material/Patients and Methods**

We developed and tested an AI-based system capable of processing and interpreting regulatory guidelines and biomedical literature to generate complete, structured dossiers. The system uses advanced NLP to extract pertinent data from clinical trials, research publications, and regulatory standards (e.g., FDA, EMA). For demonstration purposes, we simulated dossier generation for an Investigational New Drug (IND) application, using a simulated dataset of 30 study reports and related literature. The AI tool was evaluated based on the completeness, accuracy, and regulatory compliance of the generated dossier compared to manual processes.

**Results**

The AI-based system successfully generated regulatory dossiers with a significant reduction in processing time—with potential to cut dossier creation by 25-75% compared to manual methods. A regulatory expert assessed the AI-generated dossier for completeness and compliance and reported the accuracy of content and correct structuring according to regulatory authority requirements.

**Conclusion**

The demonstration shows that AI-based automation of regulatory dossier generation is both feasible and effective, offering substantial time savings and improved efficiency. This technology has the potential to transform regulatory workflows in pharmaceutical and healthcare industries by reducing manual burden and ensuring compliance with regulatory standards. Future iterations will focus on increasing adaptability to various regulatory bodies and further improving dossier accuracy.

## A38: Codification and collection of patients without determined diagnosis in ERN registries

Ch. Vossler-Wolf^1^, H. Graessner^1^

^1^University Hospital Tübingen, Institute of Medical Genetics and Applied Genomics, Tübingen, Germany

ERN-RND

**Contact**: christina.vossler-wolf@med.uni-tuebingen.de.

*Orphanet Journal of Rare Diseases 2026,* **21(1):**A38

All ERNs collect patient data in ERN registries. This might include patients without a determined diagnosis. In order to get an overview of how the different ERNs codifying and collecting patients without a determined diagnosis we have done a survey across all ERN registries.

We asked four main questions with the option to provide additional information:

1. Is your ERN registry including patients without determined diagnosis? If no: please explain why not.
2. How is a patient without a determined diagnosis codified/marked in your registry? Orphacode or other? If other: How do you do it?
3. Do you include patients that have been codified with the ORPHAcode 616,874: Rare disorder without a determined diagnosis after full investigation?
4. Do you know, if HCPs of your ERN are using the orphacode 616,874? If yes: Please provide a list of these HCPs.

We received responses from 14 ERNs (61%). Of these, nine include patients without a determined diagnosis in their respective registry. Question 2-4 were answered very heterogeneously. There are different ways to codify or mark patients without a determined diagnosis, less than half doing that with orphacodes. Only four ERNs are including patients that have been codified these patients with the orphacode 616,874 (rare disorder without a determined diagnosis after full investigation) and even just one ERN knows, if HCPs of the respective ERN are using the orphacode 616,874.

The results will help to develop strategies to harmonise and use registry data for a cross-ERN use case on patients without a determined diagnosis. The results of this use case might have impacts on healthcare and diagnostic research. The work of a respective cross-ERN working group will be fed with the survey results.

## A39: Randomization in rare disease clinical trials with group sequential designs

D. Bodden^1^, F. König^2^, R. -D. Hilgers^1^

^1^Department of Medical Statistics - RWTH Aachen University, Aachen, Germany (*presenting author*); ^2^Center for Medical Data Science, Institute of Medical Statistics, Medical University of Vienna, Vienna, Austria

*Orphanet Journal of Rare Diseases 2026,* **21(1):**A39

**Background**

Traditional clinical trial designs continue patient enrollment until a predetermined sample size is achieved, but group sequential designs allow for interim analyses and potential early stopping for efficacy or futility. This adaptability is particularly valuable in rare diseases, where patient populations are small, recruitment is slow, and maximizing efficiency with smaller sample sizes is critical. Balancing treatment allocation during and at the end of the trial impacts the performance, i.e. control of the type one error probability of the group sequential design methods like Pocock and O’Brien & Fleming. The question is whether other methods, such as the alpha spending approach by Lan & DeMets and the inverse normal combination test, show better performance. This investigation aims to evaluate the impact of unbalanced allocation caused by the randomization process in group sequential designs using the Pocock, O’Brien & Fleming, Lan & DeMets approach and the inverse normal combination test.

**Methods**

A literature review was conducted to investigate the description of the randomization and balancing aspects in studies using a group sequential design. Following this, a simulation study was performed using various group sequential designs across different sample sizes and stages in regards to different randomization procedures. Type I error probability and power were calculated for each combination of randomization procedure and group sequential design, with additional evaluations incorporating both binding and non-binding futility boundaries.

**Results**

Only 56% of group sequential trials reported the randomization procedure, and just 34% reported both the randomization procedure and its parameters. This is crucial, as the choice of randomization procedure substantially influenced power for the inverse normal test. The design showed a notable power drop when the 1:1 allocation ratio was not kept for each stage. For Lan & DeMets with O’Brian & Fleming boundaries the simulation showed similar power across all RPs except complete randomization, which performed poorly.

**Conclusions**

When a balanced allocation ratio cannot be guaranteed for each stage, the Lan & DeMets approach is preferable for small sample clinical trials due to its robustness against deviations from the 1:1 allocation ratio in each stage. While the inverse normal combination test is beneficial for rare disease clinical trials using sample size recalculation, it should carefully be used with permuted block randomization to avoid power loss. In unblinded trials, common in rare diseases, permuted block randomization presents a risk of allocation bias, as the block size—which should be a divisor of the stage sizes—can easily be predicted in group sequential designs. These challenges may be even more substantial in more complex adaptive designs, such as platform trials, which require further investigation. There is a need for better reporting of randomization methods and stopping boundaries in group sequential trials.

